# Association between triglyceride-glucose index-a body shape index and atherosclerotic cardiovascular disease, and the modification effect of dietary patterns

**DOI:** 10.3389/fnut.2026.1682636

**Published:** 2026-03-10

**Authors:** Ping Xiao, Xiao Fu, Haiwei Rao, Xia Tao, Xin Luo

**Affiliations:** 1 Department of General Practice, The Second Affiliated Hospital, Jiangxi Medical College, Nanchang University, Nanchang, China; 2 Department of Intensive Care Unit, The Second Affiliated Hospital, Jiangxi Medical College, Nanchang University, Nanchang, China; 3 Department of Emergency Internal Medicine, The Second Affiliated Hospital, Jiangxi Medical College, Nanchang University, Nanchang, China

**Keywords:** a body shape index, atherosclerotic cardiovascular disease, cross-sectional study, triglyceride-glucose index, triglyceride-glucose index-a body shape index

## Abstract

**Background:**

The association of the triglyceride-glucose index-a body shape index (TyG-ABSI), a novel indicator integrating glucose and lipid metabolism with obesity status, with atherosclerotic cardiovascular disease (ASCVD), and the underlying mechanisms, have not been elucidated.

**Methods:**

We conducted a retrospective cross-sectional study using data from the health examination center of Nanchang University Second Affiliated Hospital. The multivariable logistic regression models, restricted cubic spline (RCS) regression models, receiver operating characteristic (ROC) curve analysis, subgroup analyses, mediation analyses, and sensitivity analyses were used to evaluate the potential relationship and explore potential mechanisms between TyG-ABSI and ASCVD.

**Results:**

Individual TyG and ABSI were both positively associated with ASCVD, and TyG and ABSI had a significantly synergistic effect on ASCVD. Furthermore, TyG-ABSI was significantly linear and positively associated with ASCVD. In the fully adjusted model, each unit increment in TyG-ABSI was associated with a 152.2% higher odds ratio of ASCVD (OR = 2.522, 95% CI: 1.388–4.054). This positive association was robust in most subgroups. In addition, TyG-ABSI presented better diagnostic performance for ASCVD diagnosis compared with TyG, ABSI, TyG-BMI, TyG-WC, and TyG-MHtR. In addition, inflammation-related indicators (CRP, SIRI, MLR, and NLR) and the oxidative stress-related indices (GGT, UA) had significant mediation effects on the association between TyG-ABSI and ASCVD. Some dietary patterns, such as aMed, HEI-2020, DASH, may have modifying effects on the association.

**Conclusions:**

This study elucidates the significant positive dose-response linear relationship of TyG-ABSI with ASCVD, the moderately diagnostic performance of TyG-ABSI for ASCVD, and the mediation effects of CRP, SIRI, MLR, NLR, GGT, and UA. However, these inflammation and oxidative indicators should be viewed strictly as exploratory and hypothesis-generating for a mediating role, due to our cross-sectional design. Dietary patterns, such as aMed, HEI-2020, DASH, may have modifying effects on the association.

## 1 Introduction

Atherosclerotic cardiovascular disease (ASCVD) encompasses coronary artery disease, cerebrovascular disease, and peripheral artery disease. It is the predominant contributor to the morbidity and mortality of cardiovascular disease worldwide (1, 2). In the USA, ASCVD accounts for 28%−43% of cardiovascular disease mortality (3, 4). Although there are no exact risk factors and etiological mechanisms for the occurrence and progression of ASCVD, more and more evidence has demonstrated that glucose and lipid metabolic levels and obesity status may be important risk factors to increase the events of cardiovascular disease (5, 6). A cohort study from China demonstrated that a high triglyceride-glucose index (TyG), as a marker of glucose and lipid metabolic levels, was positively associated with the risk of cardiovascular disease (7). In addition, emerging studies reveal that obesity is also associated with the risk of cardiovascular disease, such as heart failure (8). Epidemiological evidence demonstrates that for each 5 kg/m^2^ increase in BMI, the risk of heart failure increases by 30%−40%. Currently, studies on the association of glucose and lipid metabolic levels and obesity status with ASCVD are limited. Therefore, it is imperative to explore the relationship between glucose and lipid metabolic levels and obesity status and ASCVD.

When clinically assessing glucose and lipid metabolism levels, in addition to conventional indicators (such as fasting blood glucose and LDL-C), some surrogate markers can more sensitively and accurately reflect metabolic abnormalities, especially in risk stratification for cardiovascular diseases (such as ASCVD) (9). For example, the TyG index, calculated by triglycerides and glucose, effectively responds to glucose and lipid metabolic levels (10). Its clinical utility is well-documented, with extensive research linking higher TyG index levels to an increased risk, burden, and mortality of various cardiovascular diseases, including myocardial infarction and stroke. For instance, a recent large-scale cohort study confirmed that the TyG index was a strong independent predictor of all-cause and cardiovascular mortality, highlighting its significant public health implications (11, 12). In addition, evidence from a cohort also demonstrated that the diagnostic performance of TyG for the risk of cardiovascular diseases was with an AUC of 0.82 (95% CI: 0.80–0.86) (13). Furthermore, it has some advantages, such as being cost-effective compared to complex insulin resistance tests, remaining unaffected by insulin therapy, and predicting cardiovascular risk earlier (14). Therefore, the TyG index is used as a representative of glucose and lipid metabolic levels in our study. In addition, body fat (particularly visceral fat) is also closely linked with cardiovascular diseases (15). A body shape index (ABSI) calculated by waist circumference and height is a novel index to measure body fat levels, which more effectively represents visceral fat levels compared to the conventional obesity index (such as BMI). In a cross-sectional study from the USA, ABSI may offer predictive capability for cardiovascular disease mortality in US adults compared to BMI (16). Therefore, the ABSI is used as a representative of body fat levels in our study. Recently, some studies have begun to explore the confluence of metabolic dysfunction and body shape in cardiovascular risk. For instance, Zheng et al. (17) investigated the TyG index and ABSI in relation to cardiovascular disease and mortality, while Yue et al. (18) examined their joint effect of TyG-ABSI on accident stroke in early-stage cardiovascular-kidney-metabolic syndrome. However, evidence regarding their synergistic impact on the composite outcome of ASCVD remains limited, and the potential mechanisms have not been elucidated.

Emerging evidence underscores the critical effect of dietary patterns on cardiovascular disease risk (19). Some studies demonstrated that adherence to cardioprotective diets [e.g., Mediterranean, Dietary Approaches to Stop Hypertension (DASH)] is associated with a 20%−30% reduction in cardiovascular disease risk (HR = 0.72, 95% CI: 0.61–0.86) (20). Conversely, Western diets (high in refined carbs and red meat) elevate cardiovascular disease risk through oxidative stress (e.g., elevated 8-OHdG levels), insulin resistance (e.g., HOMA-IR increased by 1.5-fold), and visceral adiposity (e.g., waist circumference increased by 4–6 cm) (21). This evidence supports that dietary pattern modification may be a vital strategy in the prevention of cardiovascular disease risk. However, the studies on the interaction effect of various dietary patterns on ASCVD risk are limited, and whether dietary patterns have significant interaction effects on the association between ASCVD risk and the TyG index and ABSI.

To address this knowledge gap, we conducted a retrospective cross-sectional study using population health examination data from the health examination centers of Nanchang University Second Affiliated Hospital to examine the association of ASCVD with TyG index and ABSI and investigate the potential modification effects of various dietary patterns.

## 2 Methods

### 2.1 Study design and participants

We designed a retrospective cross-sectional study using population health examination data from June 1, 2020, to June 1, 2024, in the health examination center of Nanchang University Second Affiliated Hospital to explore the combined effects of ABSI, TyG, and ASCVD. This retrospective cross-sectional study was approved by the Ethics Committee of Nanchang University Second Affiliated Hospital (NUSAH-2025-16). Each individual voluntarily signed informed consent documents following the Declaration of Helsinki principles.

**Figure 1:**
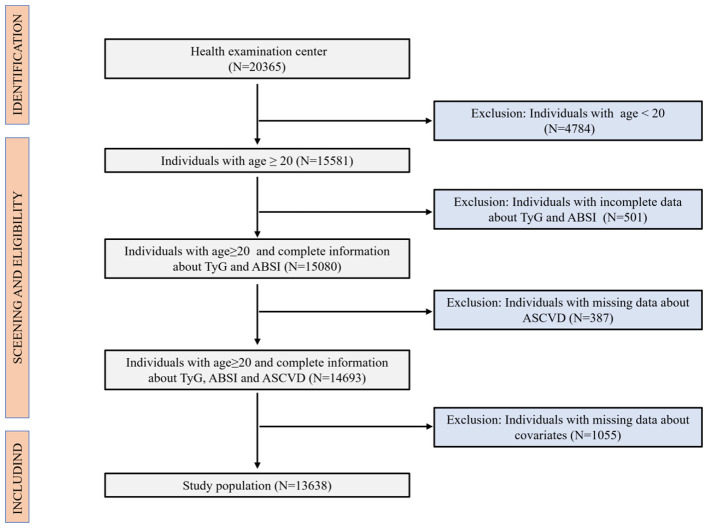
The inclusion and exclusion of the criterion.

Our cross-sectional study initially included the health examination data of 20,365 individuals from the health examination center of Nanchang University Second Affiliated Hospital. According to the inclusion and exclusion criteria of our study, individuals under 20 years of age (*N* = 4,784), those with missing TyG and ABSI data (*N* = 501), those with missing ASCVD data (*N* = 387), and those with missing covariate information (*N* = 1,055) were excluded. Finally, 13,638 individuals who met our inclusion and exclusion criteria were included in our study. A detailed flowchart (Figure 1) delineated the participant selection process.

### 2.2 Measurement of the TyG, ABSI, and TyG-ABSI

The TyG index was calculated by the following formula: TyG index = ln [fasting triglycerides (mg/dl) × fasting glucose (mg/dl)/2] (22). The ABSI was calculated by the following formula: ABSI = WC (m)/[Height (m)^1/2^ × BMI (kg/m^2^)^2/3^] (23). To explore the combined effect of TyG and ABSI, we used a novel index (TyG-ABSI). The TyG-ABSI index was calculated by the following formula: TyG-ABSI = TyG × ABSI (18).

### 2.3 Definition of ASCVD

Following the American Heart Association guidelines and established clinical criteria, ASCVD was defined as participants with physician-diagnosed cardiovascular conditions, including coronary artery disease, angina pectoris, myocardial infarction, cerebrovascular accident, or congestive heart failure (24).

### 2.4 Measurement of dietary patterns

Our dietary data was collected by using a self-reported food frequency questionnaire (FFQ) (25). Alternative mediterranean diet (aMED) typically scores based on the following nine categories of foods/nutrients, including: vegetables, fruit, whole grain, nuts, beans, fish/seafood, monounsaturated fatty acid, alcohol, red meat/processed meat. For all components except alcohol, the sex-specific median intake of our study population was used as the cut-off. 1 point was awarded to men and women whose intake was above the sex-specific median for beneficial components (vegetables, fruits, nuts, whole grains, beans, fish, and monounsaturated fatty acids) and below the sex-specific median for red and processed meat. Regarding alcohol, the component was operationalized based on total alcohol intake. 1 point was awarded to men who consumed between 10 and 25 g/day and to women who consumed between 5 and 15 g/day. Intakes outside these sex-specific ranges received 0 points. The points from all nine components were summed to create a total aMED score ranging from 0 to 9, with higher scores indicating greater adherence. Finally, 0–3 score, 4–6 score, and 7–9 score of aMed were regarded as low level, middle level, and high level (26).

The dietary approaches to stop hypertension (DASH) diet was a scientific dietary pattern aimed at lowering blood pressure and improving cardiovascular health, emphasizing high potassium, high calcium, high fiber, and low sodium intake. The DASH diet contained eight categories of foods/nutrients, including whole grains, vegetables, fruit, low-fat dairy products, red/processed meats, nuts/seeds/beans, sugar-sweetened beverages, and sodium, which was calculated following by quintile distributions within the studied population, such as components aligned with DASH recommendations (fruits, vegetables, nuts/seeds/beans, whole grains, low-fat dairy) were assigned a score from 1 (lowest quintile) to 5 (highest quintile), and components contrary to DASH recommendations (sodium, red/processed meats, sugar-sweetened beverages) were reverse-scored (1 for highest quintile, 5 for lowest quintile), resulting in a total score ranging from 8 to 40. Finally, 0–15 score, 16–30 score, and 31–40 score of DASH were regarded as low level, middle level, and high level (27).

The Dietary Inflammatory Index (DII) is a tool for quantifying the pro-inflammatory/anti-inflammatory potential of a diet, based on a score of 45 inflammation-related dietary components. The 45 dietary components assessed by DII can be divided into two broad categories: anti-inflammatory components, including dietary fiber, omega-3 fatty acids, monounsaturated fatty acids, polyphenols (such as flavonoids), vitamins A, C, D, and E, magnesium, zinc, selenium, curcumin, green tea (negative scores), and pro-inflammatory components, including saturated fatty acids, trans fatty acids, omega-6 fatty acids, refined carbohydrates, cholesterol, high GI foods, and red meat/processed meat (positive scores). Based on the total DII score ranging from approximately −8 to +8, it was stratified into three levels by tertiles: low level (−8 ≤ DII ≤ 1.3), middle level (1.3 < DII ≤ 4.6), and high level (4.6 < DII ≤ 8) (28).

The Healthy Eating Index-2020 (HEI-2020) is a dietary quality assessment tool developed by the United States Department of Agriculture (USDA) that includes 13 components to quantify dietary adherence to the Dietary Guidelines for Americans. The 13 components evaluated in HEI-2020 can be divided into two broad categories: nine categories of adequate components, including whole grains, fruits (total amount), fruits (whole fruits), vegetables (total amount), greens and beans, dairy products, protein foods (total amount), protein foods (seafood/plants), and the fatty acid ratio (the ratio of polyunsaturated and monounsaturated fatty acids to saturated fatty acids) and four categories of the limiting components, including added sugar, saturated fat, sodium, and refined grains. The HEI-2020 total score ranges from 0 to 100 points and can be divided into three levels based on the total score: low level (HEI ≤ 50), middle level (50 < HEI ≤ 80), and high level (HEI > 80) (29).

### 2.5 Covariates

Our study included the following sociodemographic, lifestyle, and clinical biomarker covariates. Sociodemographic and lifestyle covariates included age (< 60, ≥60 years), gender (female and male), education levels (less than high school, high school, more than college), and poverty index ratio (PIR; < 1.0, 1.0–3.0, and ≥3.0) (30), body mass index (BMI; < 18.5, 18.5–24.9, 25–29.9, ≥30 kg/m^2^), smoking status (never, former, current) and drinking status (never, former, current). Hypertension was defined as participants with an average of three times systolic blood pressure (SBP) ≥130 and diastolic blood pressure (DBP) ≥80 mmHg, or diagnosed by a physician, or usage of lower blood pressure medicine, which was categorized into yes or no. Diabetes was defined as participants with fasting glucose ≥126 mg/dl or HbA1c ≥6.5%, or diagnosis by a physician, or usage of lower blood glucose medicine, which was categorized into yes or no (31, 32). Clinical biomarkers covariates included waist circumference (WC), total cholesterol (TC), triglyceride (TG), low-density lipoprotein cholesterol (LDL), high-density lipoprotein cholesterol (HDL), glycated hemoglobin A1c (HbA1c), fast glucose, C-reactive protein (CRP), uric acid (UA), total cholesterol (TC), albumin (ALB), aspartate aminotransferase (AST), alanine aminotransferase (ALT), gamma-glutamyl transferase (GGT), and lactate dehydrogenase (LDH).

### 2.6 Statistical analysis

In this study, statistical analysis was performed on the baseline characteristics of the participants, using Student's *t*-test for continuous variables and χ^2^ tests for categorical variables. The TyG, ABSI, and TyG-ABSI were evaluated as both continuous and categorical variables, with quartile-based stratification (Q1–Q4) applied for categorical analyses. To assess potential associations among TyG, ABSI, TyG-ABSI, and ASCVD, we conducted multivariable logistic regression models and restricted cubic spline (RCS) regression models. To compare the diagnostic performance among TyG, ABSI, TyG-ABSI, and other related TyG indices (TyG-BMI, TyG-WC, TyG-ABSI, TyG-MHtR), for ASCVD diagnosis, receiver operating characteristic (ROC) curve analysis was used with some key indicators to represent diagnostic performance, such as area under the curve (AUC), sensitivity, specificity, positive predictive value (PPV), and negative predictive value (NPV). Furthermore, to explore the increased discrimination capacity of these indices for ASCVD, net reclassification improvement (NRI) and integrated discrimination improvement (IDI) were calculated. To assess the clinical net benefit capacity of these indices, decision curve analysis (DCA) was used. To evaluate the robustness of the association of TyG, ABSI, and TyG-ABSI with ASCVD, we conducted subgroup analyses and sensitivity analyses. Given the cross-sectional design, we used multiple serial mediation analyses to explore the potential mechanism. It is crucial to note that these analyses were explicitly exploratory and hypothesis-generating, since all analyses cannot establish causality or temporal sequence in the cross-sectional study. To address the issue of multiple comparisons and control the False Discovery Rate (FDR), we performed *post-hoc* adjustments using the Benjamini–Hochberg procedure. All statistical analyses were performed using R software (version 4.3.3). *P* < 0.05 was used as the statistical significance criterion.

## 3 Results

### 3.1 Baseline demographic characteristics of the participants

The baseline characteristics of the participants were summarized in Table 1. As shown in Table 1, this cross-sectional study included 13,638 participants aged ≥20 years, among whom 48.97% (*n* = 6,679) were female, and 29.21% (*n* = 3,983) met diagnostic criteria for ASCVD. Significant differences (*P* < 0.05) were observed across most covariates when comparing participants with ASCVD to those with non-ASCVD.

**Table 1:** Baseline demographic characteristics of the participants.

**Variables**	**Total (*n* = 13,638)**	**ASCVD (*n* = 3,983)**	**None-ASCVD (*n* = 9,655)**	***P*-value**
Age	47.91 (14.38)	50.42 (15.14)	45.33 (13.79)	<0.001
**Gender**				<0.001
Female	6,679 (48.97)	1,680 (42.18)	4,999 (51.78)	
Male	6,959 (51.03)	2,303 (57.82)	4,656 (48.22)	
**Educational levels**				<0.001
Less than high school	2,573 (18.87)	718 (18.03)	1,855 (19.21)	
High school	3,483 (25.54)	981 (24.63)	2,502 (25.91)	
College or above	7,582 (55.59)	2,284 (57.34)	5,298 (54.88)	
**PIR**				<0.001
PIR < 1	2,363 (17.32)	679 (17.05)	1,684 (17.44)	
1 ≤ PIR < 3	3,624 (26.57)	1,421 (35.67)	2,203 (22.82)	
PIR > 3	7,651 (56.11)	1,883 (47.28)	5,768 (59.74)	
**BMI**				<0.001
Underweight	710 (5.20)	265 (6.65)	445 (4.61)	
Normal weight	5,543 (40.64)	1,230 (30.88)	4,313 (44.67)	
Overweight	3,327 (24.40)	1,018 (25.56)	2,309 (23.92)	
Obesity	4,058 (29.76)	1,470 (36.91)	2,588 (26.80)	
**Drinking status**				<0.001
Never drinker	3,542 (25.97)	769 (19.31)	2,773 (28.72)	
Former drinker	2,576 (18.89)	897 (24.31)	1,679 (17.39)	
Current drinker	7,520 (55.14)	2,317 (67.52)	5,203 (53.89)	
**Smoking status**				<0.001
Never smoker	2,135 (15.65)	453 (11.37)	1,682 (17.42)	
Former smoker	4,459 (32.70)	1,007 (25.28)	3,452 (35.75)	
Current smoker	7,044 (51.65)	2,523 (63.35)	4,521 (46.83)	
**Physical activity**				<0.001
Vigorous level	4,697 (34.44)	848 (21.29)	3,849 (39.87)	
Middle level	4,241 (31.10)	1,358 (34.09)	2,883 (29.86)	
Low level	4,700 (34.46)	1,777 (44.62)	2,923 (30.27)	
**Hypertension**				<0.001
Yes	5,109 (37.46)	1,194 (29.98)	3,915 (40.55)	
No	8,529 (62.54)	2,789 (70.02)	5,740 (59.45)	
**Diabetes**				<0.001
Yes	4,323 (31.70)	1,008 (25.31)	3,315 (34.33)	
No	9,315 (68.30)	2,975 (74.69)	6,340 (65.67)	
**Chronic kidney disease**				0.056
Yes	2,689 (19.71)	883 (22.17)	1,806 (18.71)	
No	10,949 (80.29)	3,100 (77.83)	7,849 (81.29)	
TC (mg/dl)	225.08 (16.16)	226.19 (16.76)	224.25 (15.43)	0.145
TG (mg/dl)	131.25 (15.25)	135.36 (16.34)	128.47 (14.07)	<0.001
HDL (mg/dl)	38.43 (9.43)	36.49 (10.09)	41.35 (8.21)	<0.001
HbA1c (%)	5.40 (0.64)	5.76 (0.70)	5.18 (0.55)	0.089
Fast glucose (mg/dl)	93.24 (13.35)	97.43 (14.16)	90.19 (12.38)	<0.001
CRP (mg/L)	3.04 (1.89)	3.65 (2.01)	2.69 (1.28)	<0.001
UA (mg/dl)	5.87 (2.49)	6.52 (2.86)	5.25 (2.12)	<0.001
AST (U/L)	21.26 (5.18)	23.28 (5.98)	20.02 (4.85)	<0.001
ALB (g/L)	46.02 (7.42)	49.05 (8.81)	44.24 (6.06)	<0.001
ALT (U/L)	25.04 (6.06)	27.16 (7.16)	22.24 (5.17)	<0.001
GGT (U/L)	37.06 (6.56)	39.31 (7.96)	34.19 (6.09)	<0.001
LDH (U/L)	141.18 (14.21)	144.67 (15.56)	138.06 (13.89)	<0.001

Categorical variables were presented as %, with χ^2^ tests comparing statistical differences between groups. Continuous variables were reported as mean ± standard deviation (SD), with Student's t-test in between-group comparisons. *P* < 0.05 was regarded as statistically significant. PIR, family income-to-poverty ratio; BMI, body mass index; WC, waist circumference; TC, total cholesterol; TG, triglyceride; LDL, low-density lipoprotein cholesterol; HDL, high-density lipoprotein cholesterol; HbA1c, glycated hemoglobin A1c; CRP, C-reactive protein; UA, uric acid; ALB, albumin; ALT, alanine aminotransferase; AST, aspartate aminotransferase; GGT, gamma-glutamyl transferase; LDH, lactate dehydrogenase; ASCVD, atherosclerotic cardiovascular disease.

### 3.2 Association between TyG and ABSI and ASCVD events

**Figure 2:**
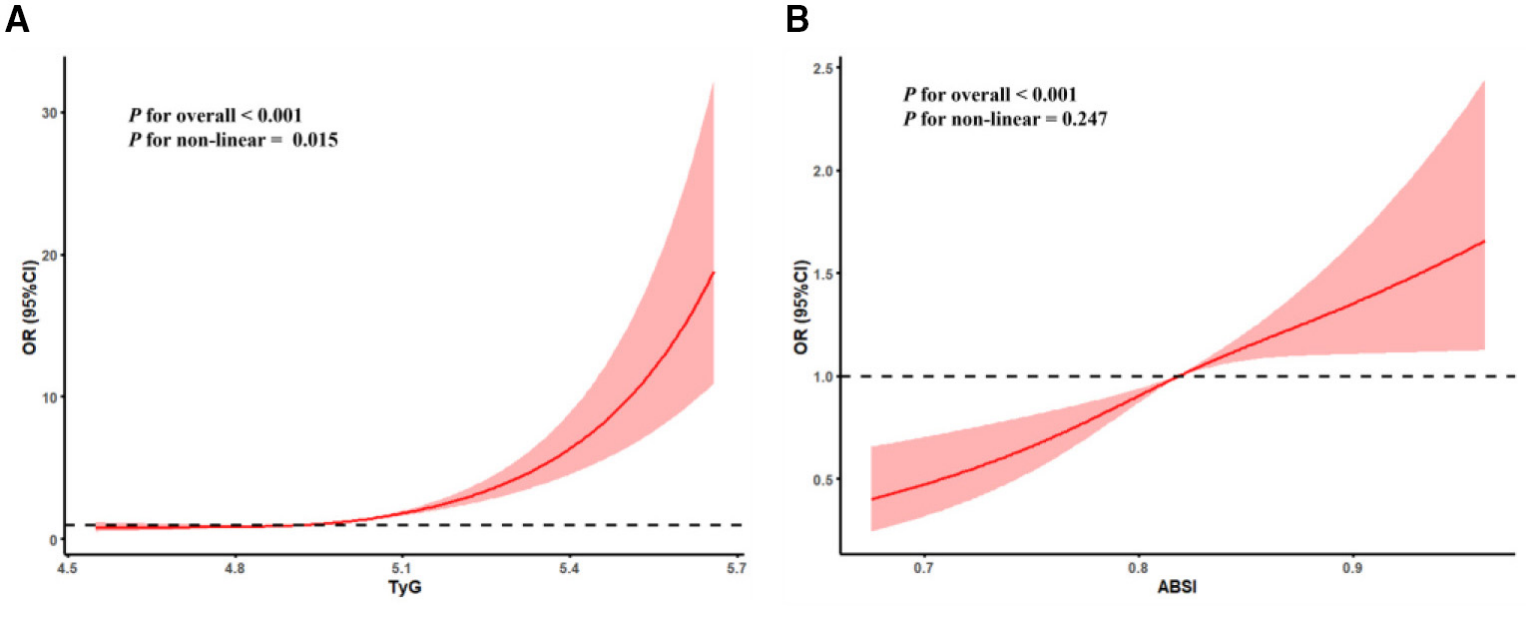
The dose-response association between TyG **(A)** and ABSI **(B)** and ASCVD.

In the cross-sectional study, the multivariable logistic models were used to explore the associations between TyG, ABSI, and ASCVD. As shown in Table 2, after adjusting all covariates, each standardized unit increase in TyG and ABSI was associated with a 136.2% (OR = 2.362, 95% CI: 2.081–3.253, *P*-value: <0.001) and 162.4% (OR = 2.624, 95% CI: 1.308–4.064, *P*-value: <0.001) higher odds of ASCVD, respectively. In addition, compared with the lowest-quartile referent TyG and ABSI group, the highest quartile groups of TyG and ABSI had a 171.4% (OR = 2.714, 95% CI: 2.214–3.526, *P*-value: < 0.001) and 60.9% (OR = 1.609, 95% CI: 1.278–2.026, *P*-value: < 0.001) higher odds of ASCVD, respectively. The RCS curves were conducted to assess the potential non-linear relationship between TyG and ABSI, and ASCVD. After adjusting all covariates, TyG (*P* for overall <0.001, *P* for non-linear = 0.015) and exhibited a non-linear dose-response relationship with OR of ASCVD, and ABSI (*P* for overall < 0.001, *P* for non-linear = 0.247) both exhibited a linear dose-response relationship with OR of ASCVD in Figure 2.

**Table 2:** Association between TyG and ABSI, and ASCVD.

	TyG		ABSI	
**Models**	**OR (95% CI)**	***P*-value**	**OR (95% CI)**	***P*-value**
**Model I**				
Continuous	3.192 (2.073, 4.914)	<0.001	3.374 (2.341, 5.604)	<0.001
Q1	Reference		Reference	
Q2	0.882 (0.710, 1.095)	0.255	1.559 (1.260, 1.932)	<0.001
Q3	1.403 (1.147, 1.717)	0.001	2.236 (1.826, 2.743)	<0.001
Q4	3.350 (2.780 4.038)	<0.001	3.227 (2.647, 3.936)	<0.001
*P* for trend	<0.001		<0.001	
**Model II**				
Continuous	3.090 (1.982, 4.814)	<0.001	3.181 (2.372, 5.880)	<0.001
Q1	Reference		Reference	
Q2	0.887 (0.711, 1.107)	0.288	1.385 (1.115, 1.721)	0.003
Q3	1.387 (1.127, 1.708)	0.002	1.787 (1.443, 2.212)	<0.001
Q4	3.353 (2.764, 4.066)	<0.001	2.284 (1.832, 2.847)	<0.001
*P* for trend	<0.001		<0.001	
**Model III**				
Continuous	2.362 (2.081, 3.253)	<0.001	2.624 (1.308, 4.064)	<0.001
Q1	Reference		Reference	
Q2	0.853 (0.681, 1.070)	0.169	1.153 (0.922, 1.442)	0.211
Q3	1.255 (1.015, 1.553)	0.036	1.387 (1.113, 1.729)	0.003
Q4	2.714 (2.214, 3.526)	<0.001	1.609 (1.278, 2.026)	<0.001
*P* for trend	0.006		<0.001	

Q1 was regarded as the reference group. Model I: crude model. Model II: adjusted for age, gender, education levels, and PIR based on Model I. Model III: adjusted for all covariates, except for CRP, GGT, UA, SIRI, NLR, and MLR. *P* < 0.05 was regarded as statistically significant. Q, quartile; OR, odds ratio.

### 3.3 Association between the combined effect of TyG and ABSI and ASCVD

**Figure 3:**
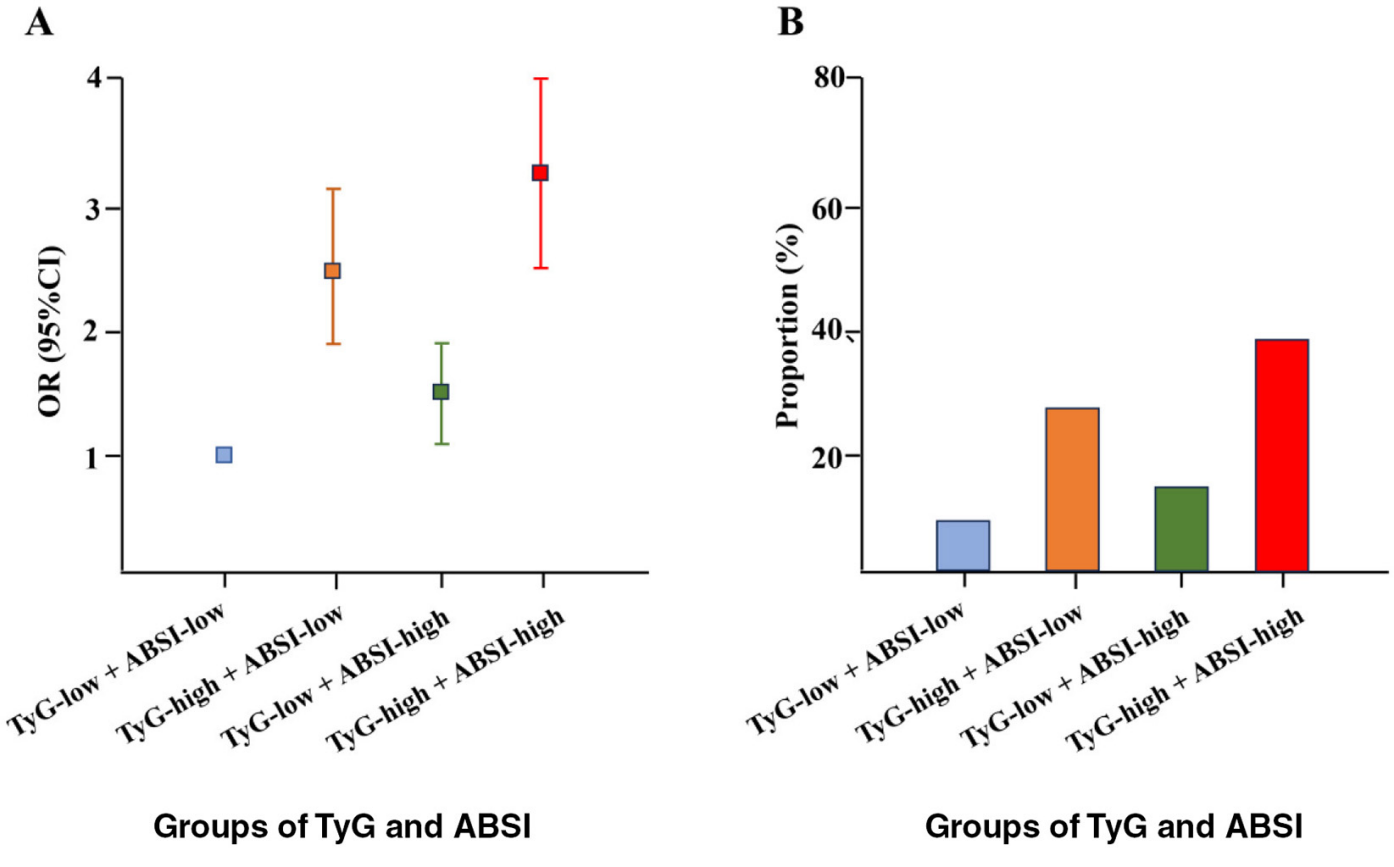
Association **(A)** and proportion **(B)** between the combined effect of TyG and ABSI and ASCVD.

To explore the association between the combined effect of TyG and ABSI and ASCVD, TyG and ABSI were both stratified into two levels based on the median value (low level and high level), and the TyG-low and ABSI-low group was regarded as the referent group. As shown in Table 3, after adjusting all covariates, compared with the TyG-low and ABSI-low group, the TyG-low and ABSI-high group, the TyG-high and ABSI-low group, and the TyG-high and ABSI-high group had 41.2% (OR = 1.412, 95% CI: 1.072–1.919, *P*-value: < 0.001), 148.5% (OR = 2.485, 95% CI: 1.829–3.126, *P*-value: < 0.001), and 224.6% (OR = 3.246, 95% CI: 2.686–4.022, *P*-value: < 0.001) higher odds of ASCVD, respectively. In addition, the visualized results of the association between the combined effect of TyG and ABSI and ASCVD were presented in Figure 3.

**Table 3:** Association of the combined effect of TyG and ABSI with ASCVD risk.

**Groups**	***N* = ASCVD (%)**	**OR (95% CI)**	***P*-value**
TyG-low + ABSI-low	489 (12.27)	Reference	Reference
TyG-high + ABSI-low	1,222 (30.68)	2.485 (1.829, 3.126)	<0.001
TyG-low + ABSI-high	684 (17.17)	1.412 (1.072, 1.919)	0.014
TyG-high + ABSI-high	1,588 (39.88)	3.246 (2.686, 4.022)	<0.001

The group of TyG-low + ABSI-low was regarded as the reference group. TyG-low < 4.78, TyG-high ≥ 4.78, ABSI-low < 0.81, ABSI-high ≥ 0.81. All covariates were adjusted in the model, except for CRP, GGT, UA, SIRI, NLR, and MLR. *P* < 0.05 was regarded as statistically significant.

### 3.4 Association between TyG-ABSI and ASCVD events

**Figure 4:**
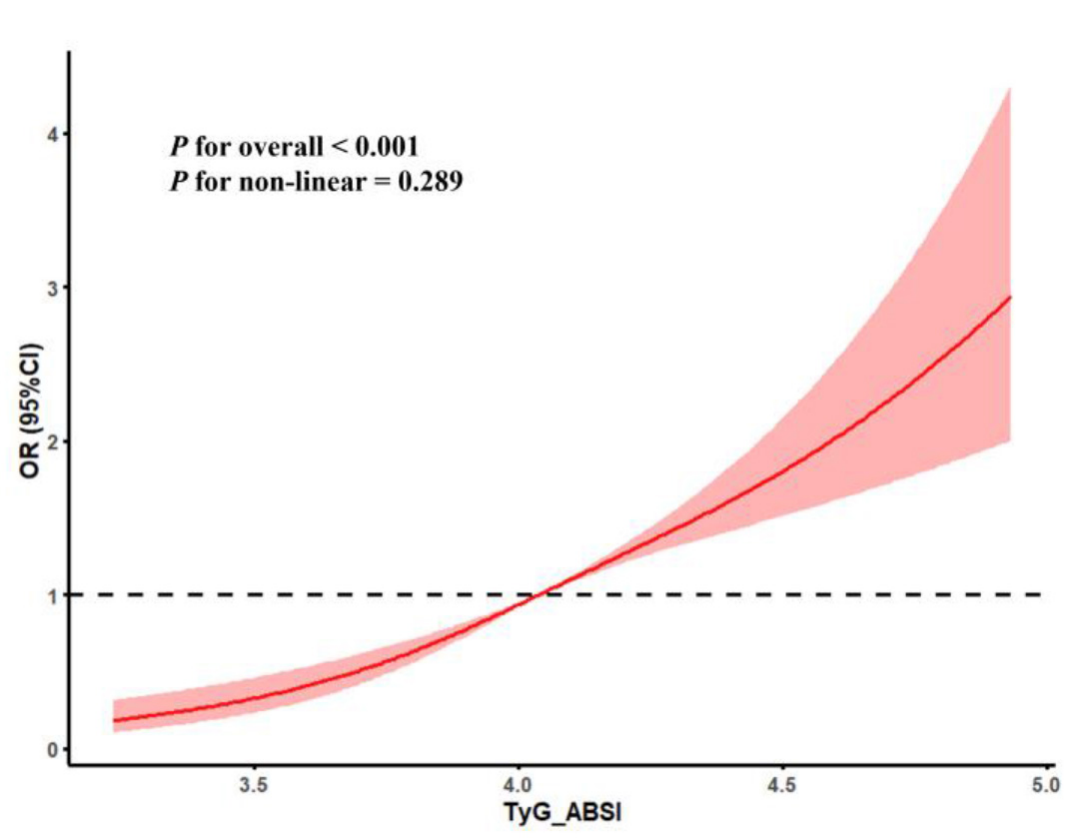
The linear dose-response association between TyG-ABSI and ASCVD.

In this study, since the above result demonstrated that TyG and ABSI were both positively associated with ASCVD, we used an index, TyG-ABSI, calculated by TyG multiplied by ABSI, to simultaneously represent the combined TyG and ABSI levels. As shown in Table 4, after adjusting all covariates, each standardized unit increase in TyG-ABSI was associated with a 152.2% (OR = 2.522, 95% CI: 1.388–4.054, *P*-value: < 0.001) higher odds of ASCVD. In addition, compared with the lowest-quartile referent TyG-ABSI group, the highest-quartile group had a 179.2% (OR = 2.792, 95% CI: 1.555–4.209, *P*-value: < 0.001) higher odds of ASCVD after adjusting all covariates. The RCS curves demonstrated that after adjusting all covariates, TyG-ABSI had a linear dose-response relationship with ASCVD in Figure 4 (*P* for overall < 0.001, *P* for non-linear = 0.289).

**Table 4:** Association between TyG-ABSI and ASCVD.

**Models**	**OR (95% CI)**	***P*-value**
**Model I**		
Continuous	3.087 (2.110, 5.162)	<0.001
Q1	Reference	
Q2	1.124 (1.062, 1.310)	<0.001
Q3	1.771 (1.371, 2.743)	<0.001
Q4	2.966 (1.775, 4.454)	<0.001
*P* for trend	<0.001	
**Model II**		
Continuous	3.159 (1.478, 5.356)	<0.001
Q1	Reference	
Q2	1.187 (1.076, 1.377)	<0.001
Q3	1.625 (1.415, 2.940)	<0.001
Q4	2.805 (1.783, 4.102)	<0.001
*P* for trend	<0.001	
**Model III**		
Continuous	2.522 (1.388, 4.054)	<0.001
Q1	Reference	
Q2	1.163 (1.078, 1.575)	<0.001
Q3	1.416 (1.392, 2.080)	<0.001
Q4	2.792 (1.555, 4.209)	<0.001
*P* for trend	<0.001	

Q1 was regarded as the reference group. All covariates were adjusted in the model, except for CRP, GGT, UA, SIRI, NLR, and MLR. *P* < 0.05 was regarded as statistically significant. Q, quartile; OR, odds ratio.

### 3.5 Subgroup analyses

In this study, to assess the consistency of the positive association between TyG-ABSI and ASCVD, we conducted subgroup analyses stratified by key sociodemographic, lifestyle, and comorbidity characteristics, including age, gender, ethnicity, educational levels, poverty index ratio, drinking status, and hypertension, etc. As detailed in Table 5, positive associations between TyG-ABSI and ASCVD remained present across most subgroups after adjusting for all covariates. For example, when stratified by age into two subgroups (< 60 and ≥60 years), each standardized unit elevate in TyG-ABSI of the two subgroups was significantly assocaitied with a 195.2% (OR = 2.952, 95% CI: 2.390–4.235, *P*-value: < 0.001) and 118.1% (OR = 2.181, 95% CI: 1.272–3.084, *P*-value: < 0.001) higher odds of ASCVD, respectively. Notably, subgroup analyses also revealed no significant effect modification in these subgroups on the association between TyG-ABSI and ASCVD (all *P* for interaction >0.05).

**Table 5:** Association between TyG-ABSI and ASCVD in various subgroups.

**Subgroups**	**OR (95% CI)**	***P*-value**	***P*-for interaction**
**Age**			0.673
< 60 years	2.952 (2.390, 4.235)	<0.001	
≥60 years	2.181 (1.272, 3.084)	<0.001	
**Gender**			0.486
Female	2.685 (2.121, 3.245)	<0.001	
Male	2.240 (1.791, 2.945)	<0.001	
**Educational levels**			0.319
Less than high-school	3.182 (2.041, 4.576)	<0.001	
High school	2.474 (1.651, 3.621)	<0.001	
College or above	2.053 (1.351, 3.146)	<0.001	
**Poverty index ratio**			0.659
PIR < 1	2.159 (1.541, 3.394)	<0.001	
1 ≤ PIR < 3	3.048 (2.161, 4.462)	<0.001	
PIR ≥ 3	2.490 (1.881, 3.636)	<0.001	
**BMI**			0.742
Underweight	3.213 (2.453, 4.539)	<0.001	
Normal weight	2.081 (1.381, 3.264)	<0.001	
Overweight	2.241 (1.782, 3.350)	<0.001	
Obesity	2.781 (2.008, 3.995)	<0.001	
**Drinking status**			0.568
Never	2.481 (1.752, 3.569)	0.009	
Former	2.003 (1.421, 3.181)	<0.001	
Current	3.235 (2.461, 4.368)	<0.001	
**Smoking status**			0.581
Never	2.071 (1.502, 3.154)	<0.001	
Former	2.339 (1.742, 3.583)	<0.001	
Current	2.922 (2.043, 4.133)	<0.001	
**Physical activity**			0.648
Vigorous level	1.681 (1.142, 2.521)	<0.001	
Middle level	2.422 (1.693, 3.423)	<0.001	
Other levels	3.244 (2.295, 4.488)	<0.001	
**Hypertension**			0.713
Yes	3.014 (2.412, 3.864)	<0.001	
No	2.242 (1.612, 3.612)	<0.001	
**Diabetes**			0.698
Yes	2.972 (2.121, 3.843)	<0.001	
No	1.956 (1.262, 3.202)	<0.001	
**Chronic kidney disease**			0.742
Yes	2.665 (2.140, 3.326)	<0.001	
No	2.405 (1.813, 3.415)	<0.001	

All covariates were adjusted in the model, except for CRP, GGT, UA, SIRI, NLR, and MLR. *P* < 0.05 was regarded as statistically significant.

### 3.6 Increased diagnostic effect of TyG-ABSI on the ASCVD

**Figure 5:**
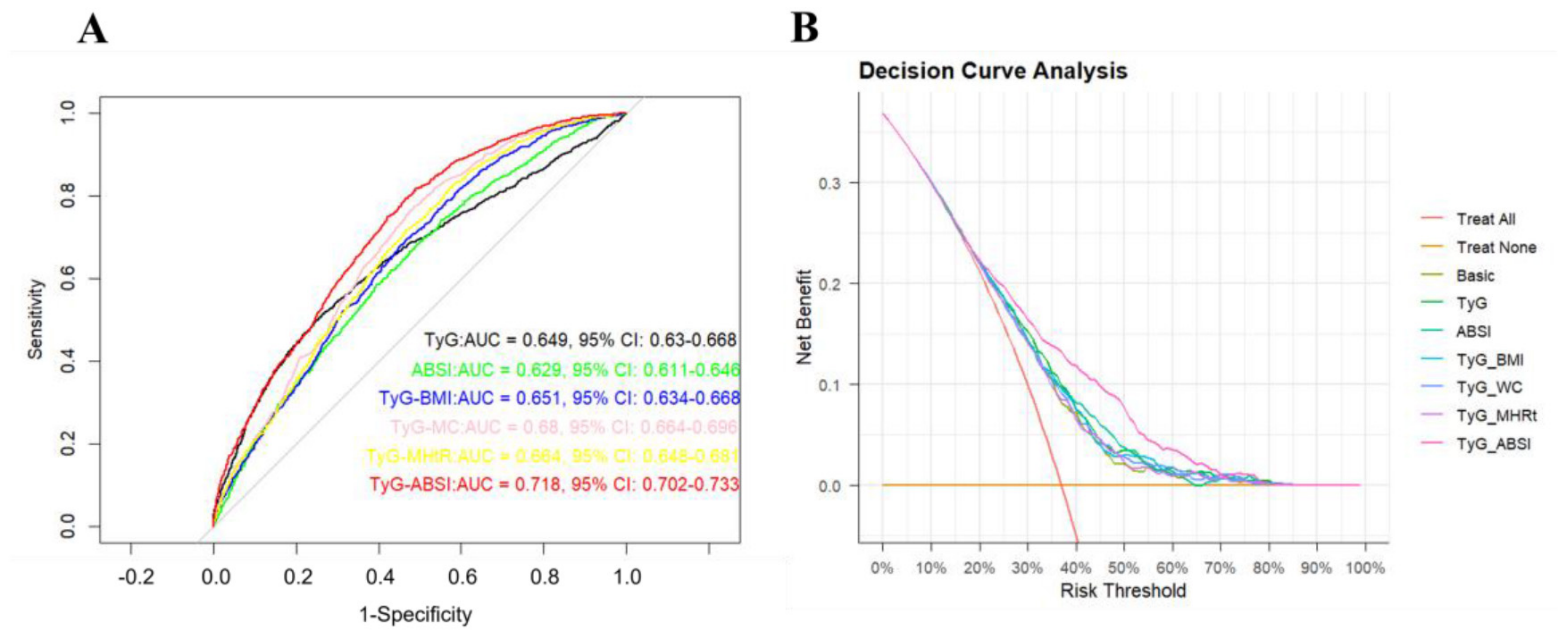
The diagnostic performance of related TyG indices for the ASCVD. **(A)** The ROC curve for the diagnostic performance of related TyG indices for ASCVD in multivariable logistic regression models. **(B)** The DCA curve for the clinical net benefit of related TyG indices for ASCVD in multivariable logistic regression models.

Emerging evidence has established that TyG-related indicators, such as TyG, TyG-BMI, and TyG-MC, were associated with ASCVD (33–38). However, the diagnostic utility of TyG-ABSI, as a combined indicator of TyG and ABSI levels, for the ASCVD remained unclear. Therefore, in this study, we employed the ROC curve and some related key indicators of diagnostic performance (AUC, sensitivity, specificity, accuracy, PPV, and cut-off value) to assess the diagnostic capacity of TyG-ABSI on the ASCVD in multivariable logistic regression models. The multivariable logistic regression models included a basic model (the basic model was referred to as all covariates were adjusted in this model, except for CRP, GGT, UA, SIRI, NLR, and MLR) and extended models (the extended models were referred to as related TyG indices, such as TyG, TyG-BMI, TyG-MC, TyG-MHtR, and TyG-ABSI, which were added into this basic model). As shown in Figure 5A and Supplementary Table S1, our results revealed that TyG-ABSI presented a moderate diagnostic performance for ASCVD, with an AUC (0.718; 95% CI, 0.702–0.733), sensitivity (0.726), specificity (0.618), and accuracy (0.678) compared with other TyG-related indicators in these extended models. In addition, we used the IDI and NRI to explore TyG-related indicators discrimination for the ASCVD. Our result showed that TyG-ABSI had the highest discrimination capacity for ASCVD among these TyG-related indicators with IDI (0.095, 95% CI: 0.076–0.178, *P*-value: < 0.001) and NRI (0.078, 95% CI: 0.045–0.113, *P*-value: < 0.001) in these extended models (Table 6). In addition, the result of DCA also showed that TyG-ABSI had a higher clinical net benefit capacity compared to other related TyG indices in these extended models (Figure 5B), which was consistent with our main results. The above results suggest TyG-ABSI had a superior diagnostic performance for ASCVD.

**Table 6:** Discrimination performance of the ASCVD diagnosis.

**Models**	**NRI (95% CI)**	***P*-value**	**IDI (95% CI)**	***P*-value**
Basic model	Reference		Reference	
TyG + basic model	0.030 (0.008, 0.051)	<0.001	0.018 (0.012, 0.027)	<0.001
ABSI + basic model	0.025 (−0.003, 0.044)	0.071	0.011 (−0.005, 0.029)	0.084
TyG-BMI + basic model	0.037 (0.014, 0.062)	<0.010	0.024 (0.010, 0.041)	<0.001
TyG-WC + basic model	0.024 (0.010, 0.046)	<0.001	0.009 (0.001, 0.023)	0.001
TyG-MHtR + basic model	0.041 (0.019, 0.074)	<0.001	0.027 (0.014, 0.048)	<0.001
TyG-ABSI + basic model	0.115 (0.076, 0.178)	<0.001	0.078 (0.045, 0.113)	<0.001

Basic model was refered the model that involved all covariates, except for CRP, GGT, UA, SIRI, NLR, and MLR, and this model was regarded as the reference group. *P* < 0.05 was regarded as statistically significant. NRI, net reclassification improvement; IDI, integrated discrimination improvement.

### 3.7 The mediation effects of inflammatory and oxidative indicators

**Figure 6:**
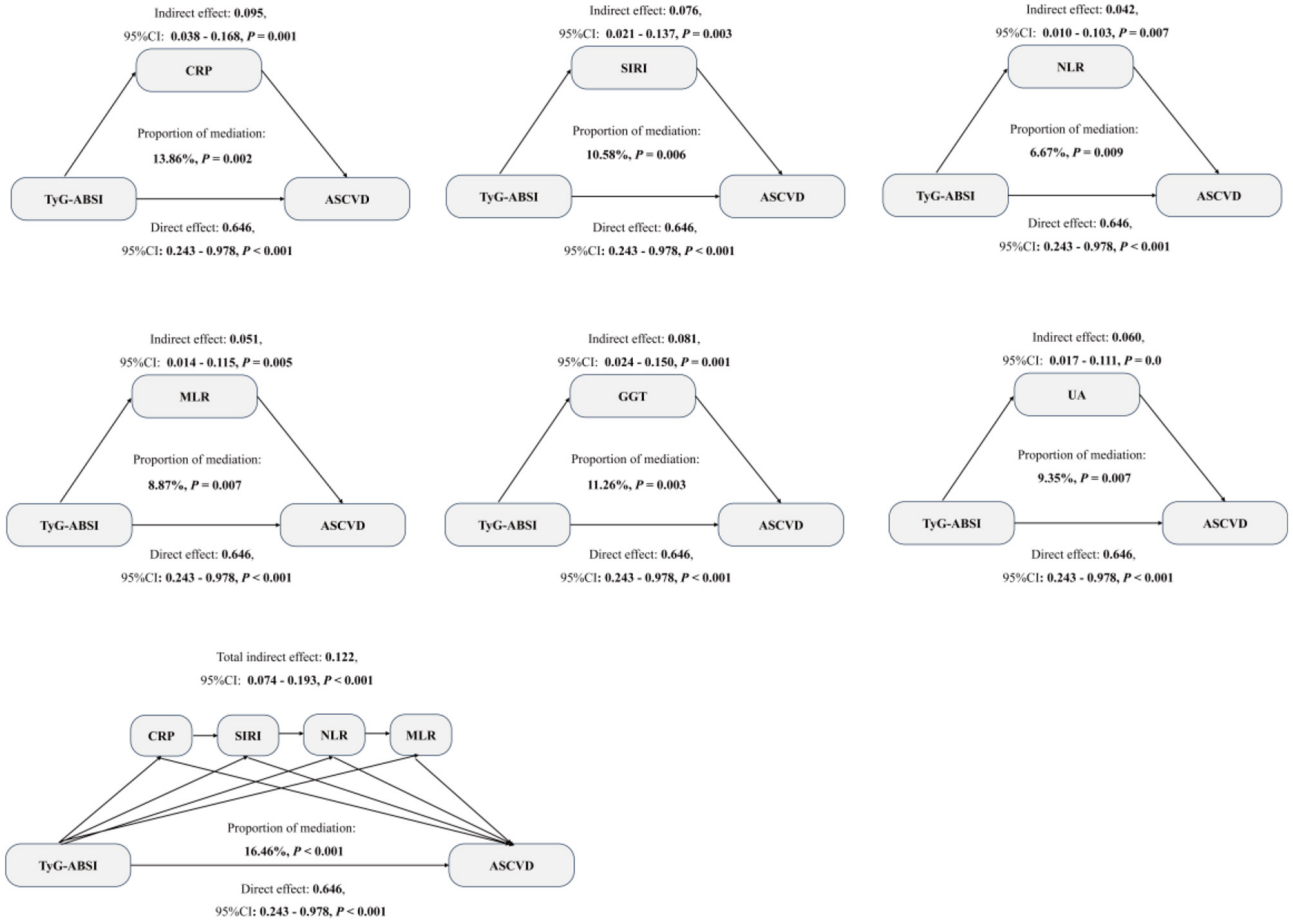
Mediation analysis was used to show the mediation effects of CRP, SIRI, NLR, MLR, GGT, and UA. All covariates were adjusted in the model, except for CRP, GGT, UA, SIRI, NLR, and MLR.

Some studies have demonstrated that inflammatory and oxidative indicators, such as C-reactive protein (CRP), systemic inflammatory response index (SIRI), neutrophil-to-lymphocyte ratio (NLR), monocyte-to-lymphocyte ratio (MLR), gamma-glutamyl transferase (GGT), and uric acid (UA), were positively associated with ASCVD (39–41). In exploratory, we used cross-sectional mediation analyses to examine the extent to which the observed associations between TyG-ABSI and ASCVD were statistically accounted for by these inflammatory and oxidative indicators. First, we explore the association between TyG-ABSI and CRP, SIRI, NLR, MLR, GGT, and UA. As detailed in Supplementary Table S2, a significant positive association of TyG-ABSI with CRP, SIRI, NLR, MLR, GGT, and UA was observed (all *P* < 0.05). Then, the association of CRP, SIRI, NLR, MLR, GGT, and UA with ASCVD was assessed. Our results showed that these inflammatory and oxidative indicators were positively associated with ASCVD in Supplementary Table S3 (all *P* < 0.05). Finally, the result of the mediation analysis illustrated that significant mediation effects of CRP, SIRI, NLR, MLR, GGT, and UA on the association between TyG-ABSI and ASCVD, with the mediated proportion of 13.56, 10.58, 6.67, 8.87, 11.26, and 9.35%, respectively (Figure 6). In addition, some previous studies demonstrated that CRP, SIRI, NLR, and MLR may have potential sequential biological relationships among them (42). Therefore, to further control the potential biological overlap of these inflammatory indicators, a serial multiple mediation analysis was conducted. Our results showed that the total indirect effect of TyG-ABSI on ASCVD through the inflammatory biomarker chain was significant (β = 0.122, 95% CI: 0.074–0.193, *P* < 0.001), accounting for 16.64% of the association in the specified serial mediation model (Figure 6). Our exploratory mediation analyses indicated that a substantial proportion of the association between TyG-ABSI and ASCVD was jointly accounted for by inflammatory and oxidative indicators, and provided a pluasibel hypotheses to validate in future longitudinal research.

### 3.8 The modification effect of four dietary patterns

Some evidence has shown the essential role and potential mechanisms of dietary patterns in the prevention of ASCVD prevalence, such as anti-inflammatory, antioxidant, and metabolic regulation (43, 44). Since dietary patterns represent the overall dietary structure rather than focusing on individual nutrients, dietary pattern adjustment may be a more effective way to reduce the prevalence of ASCVD. To explore the potential effect of dietary patterns on the association between TyG-ABSI and ASCVD, we compared the interaction performance of four dietary patterns (aMed, DII, HEI-2020, and DASH) stratified into three levels (low level, middle level, and high level). As shown in Table 7, our results revealed that high levels of aMed (OR = 0.675, 95% CI: 0.472–0.887, *P-*value: < 0.001), HEI-2020 (OR = 0.836, 95% CI: 0.686–0.976, *P*-value: < 0.001), and DASH (OR = 0.769, 95% CI: 0.568–0.918, *P*-value: < 0.001) had significantly lower prevalence of ASCVD induced by TyG-ABSI. In addition, the results of interaction tests revelaed that aMed, HEI-2020, and DASH may have a significant modification effect on the association between TyG-ABSI and ASCVD (all *P* for interaction < 0.001). However, DII had no modification effect on the association (*P* for interaction = 0.489). After adjusting for FDR, the main results remained consistent.

**Table 7:** Association of TyG-ABSI with ASCVD in various levels of four dietary patterns

**Dietary patterns**	**OR (95% CI)**	***P*-value**	***P-*adjusted**	***P* for interaction**
**aMed**				<0.001
Low level	2.892 (1.934, 4.013)	<0.001	<0.001	
Middle level	1.556 (1.174, 2.256)	<0.001	<0.001	
High level	0.675 (0.472, 0.887)	<0.001	<0.001	
**DII**				0.489
Low level	2.753 (1.167, 2.789)	<0.001	<0.001	
Middle level	3.368 (2.479, 4.014)	<0.001	<0.001	
High level	3.955 (2.723, 5.386)	<0.001	<0.001	
**HEI-2020**				<0.001
Low level	2.683 (1.231, 3.145)	<0.001	<0.001	
Middle level	1.427 (1.079, 2.056)	<0.001	<0.001	
High level	0.836 (0.686, 0.976)	<0.001	<0.001	
**DASH**				<0.001
Low level	2.512 (1.321, 2.675)	<0.001	<0.001	
Middle level	1.698 (1.279, 2.346)	<0.001	<0.001	
High level	0.769 (0.568, 0.918)	<0.001	<0.001	

All covariates were adjusted in the model, except for CRP, GGT, UA, SIRI, NLR, and MLR. *P*-value was regarded as the crude *P*-value. *P*-adjusted was regarded as a *P*-value adjustment using the Benjamini–Hochberg procedure. *P* < 0.05 was regarded as statistically significant.

### 3.9 Sensitivity analysis

To further explore the association between TyG-ABSI and ASCVD, we performed some sensitivity analyses. Some studies have demonstrated that women's menopausal status and medication use (e.g., anti-diabetics, anti-inflammatories, antihypertensives, and statins) may be associated with the occurrence and progression of ASCVD. To minimize the potential effect of these factors on the association between TyG-ABSI and ASCVD, we additionally added these factors into our models based on previously included factors to control their confounding effect. Our results showed that after including these factors, the association between TyG-ABSI and ASCVD remained positive, which was consistent with our main results (Supplementary Tables S4, S5). To reduce the potential scale dominance of TyG-ABSI construction, calculated by the multiplication of TyG and ABSI, on the association, we standardized TyG and ABSI before multiplication. Our results showed that after standardized TyG and ABSI, the positive association of TyG-ABSI with ASCVD remained robust (Supplementary Table S6).

## 4 Discussion

In this study, we designed a retrospective cross-sectional study to explore the association between TyG-ABSI and ASCVD and the potential mechanism. Our results showed that TyG and ABSI were both positively and linearly associated with ASCVD. In addition, TyG and ABSI had a synergistic effect on increased ASCVD prevalence. Thus, we used an index, TyG-ABSI, to simultaneously represent the combined TyG and ABSI levels, and further explore the association between TyG-ABSI and ASCVD. Our results also revealed that TyG-ABSI was positively and linearly associated with ASCVD. The association was robust in all subgroups. TyG-ABSI was superior in diagnosis performance for ASCVD compared to TyG, ABSI, and other TyG-related indices. In addition, our results showed that inflammatory and oxidative indicators (CRP, SIRI, NLR, MLR, GGT, and UA) had significant mediation effects on this association, and some dietary patterns, such as aMed, HEI-2020, and DASH, may significant modifying effect on the association.

Epidemiological data and clinical evidence have demonstrated a close association between abnormal glucose and lipid levels and obesity status and the events of cardiovascular disease, and high levels of surrogate clinical markers of glucose and lipid levels and obesity status, such as TyG, BMI, TyG-BMI, TyG-WC, TyG-MHtR, are positively associated with elevated cardiovascular disease risk (45). Mechanically, abnormal glucose and lipid levels and obesity status could promote the formation of a state of chronic low-grade inflammation (meta-inflammation) and oxidative stress and mitochondrial damage in the body to drive ASCVD progress, including increased secretion of various inflammatory factors such as NLRP3 inflammasome, IL-1β, IL-18, and IL-6, and enhanced infiltration of M1-type macrophages (pro-inflammatory) in adipose tissue and vascular walls to amplify inflammatory signals, increased lipid peroxidation products (such as MDA and 4-HNE) promoting mitochondrial dysfunction in vascular endothelial cells and accelerating the accumulation of ROS (46, 47). In addition, it also increases endothelial dysfunction by decreasing the bioavailability of nitric oxide (NO) and increasing endothelial permeability to further improve cardiovascular disease risk (48). Currently, the combined effect of glucose and lipid levels and obesity status on ASCVD remains unclear. Therefore, we use the TyG-ABSI indicator to respond to the combined effect of glucose and lipid levels and obesity status, and explore the association of this surrogate marker with ASCVD.

In this study, we used population health examination data from Nanchang University Second Affiliated Hospital to design a retrospective cross-sectional study to investigate the relationship of individual TyG and ABSI markers and the combined effect of TyG and ABSI (TyG-ABSI) with ASCVD. Our results showed that individual TyG and ABSI markers are both positively linked with ASCVD, which was consistent with findings from Pavanello et al.'s (49) large retrospective cohort study and Gu et al.'s (50) large cross-sectional study. Pavanello et al.'s (49) study revealed that high TyG levels were significantly associated with increased incidence of ASCVD events in general adults. Additionally, the obesity-related index (ABSI) was associated with ASCVD in Gu et al.'s study (50). In the mechanism, high TyG levels may cause a persistent hyperglycemia status, which promotes activation of protein kinase C (PKC) and advanced glycation end products (AGEs), damaging vascular endothelial function. In addition, it leads to increased lipolysis and free fatty acids (FFAs) flood into the liver, promotes synthesis of very low-density lipoproteins (VLDL), and elevated levels of triglycerides (TG) and small dense LDL (sdLDL) in the circulation, accrelating the formation of fatty plaques in blood vessels, which are the basic of ASCVD progression (51).

Then, we further explore the combined effect of TyG and ABSI on ASCVD and the relationship of TyG-ABSI with ASCVD. Our results showed that the participants with combined high TyG and high ABSI levels had a more significant ASCVD prevalence compared to the participants with a simple high TyG level, or high ABSI level, or combined low TyG and low ABSI levels, indicating TyG and ABSI may have a synergistic effect on increasing ASCVD prevalence. Therefore, to further investigate the relationship of TyG and ABSI with ASCVD, we used TyG-ABSI to respond to the status of joint TyG and ABSI levels. Our findings showed that TyG-ABSI was positively and linearly associated with ASCVD. Additionally, this positive relationship maintains consistency in various subgroups, suggesting the association is stable regardless of population characteristics. Currently, there are biological mechanisms to support our findings. Since elevated TyG promotes hepatic VLDL synthesis, increasing circulating TG and sdLDL, and elevated ABSI levels increase visceral fat to release free fatty acids (FFA) and hepatic lipid deposition (fatty liver). Therefore, the “triple burden” of blood glucose, TG, and FFA may accelerate the formation and progression of atherosclerosis (52).

Currently, some evidence has demonstrated that glucose and lipid-related indices, such as TyG and TyG-MHtR, and obesity-related indices, such as BMI, WC, and ABSI, were associated with ASCVD (53, 54). For example, Zhao et al.'s (55) study demonstrated that TyG was a strong and independent predictor of CVD in two nationally representative cohorts. In another study from Pithová et al. (56), their findings showed that in patients with metabolic syndrome, ABSI was more positively associated with carotid intima-media thickness (cIMT) than BMI and waist circumference (AUC = 0.712), highlighting the superior diagnostic effect of ABSI on CVD. However, the study of the relationship between TyG-ABSI and ASCVD was limited, and the diagnostic performance of TyG-ABSI for ASCVD remained unclear. Therefore, our study explored the diagnostic performance of TyG-ABSI on ASCVD using ROC analysis, and other key indicators of diagnostic performance (sensitivity, specificity, cut-off value, NPV, and PPV), indicators of discrimination of ASCVD (IDI and NRI), and DCA analysis. Our results revealed that compared to TyG and ABSI and other TyG-related indices (TyG-BMI, TyG-WC, TyG-MHtR), the TyG-ABSI had a better diagnostic effect on ASCVD, suggesting TyG-ABSI may be a more valuable diagnostic indicator for ASCVD with a higher AUC value and higher discrimination capacity of ASCVD. TyG-ABSI is posited to associate positively with ASCVD through a confluence of interlinked pathophysiological pathways. The high TyG index, reflecting underlying insulin resistance and hyperinsulinemia, directly fosters a pro-atherogenic state (57). This includes promoting endothelial dysfunction by reducing nitric oxide bioavailability, stimulating vascular smooth muscle cell proliferation, and enhancing hepatic production of atherogenic lipoproteins (e.g., small dense LDL particles) while lowering HDL cholesterol (58). Concurrently, a high ABSI, indicative of central adiposity, is not merely a passive fat storage depot but an active endocrine organ that secretes a plethora of pro-inflammatory cytokines (e.g., TNF-α, IL-6) (59). This creates a state of chronic low-grade inflammation that damages the vascular endothelium and exacerbates systemic insulin resistance. Therefore, the TyG-ABSI composite acts as an integrated biomarker that captures the maladaptive interplay between dysfunctional metabolism and adverse body composition, providing a more comprehensive gauge of cardiovascular events than either marker alone.

In addition, some evidence has shown that abnormal glucose and lipid levels may promote inflammation and oxidative stress (60). A study compared 90 plasma biomarkers between 80 obese (BMI >30) and normal weight (BMI < 25) individuals and revealed a significant increase of inflammatory factors (IL-6, TNF-α, CRP) and oxidative stress markers (ox-LDL) in the obese group (61). In addition, a continuously rising OGTT curve (indicating abnormal glucose and lipid levels and a risk of diabetes) is significantly associated with elevated CRP, elevated PAI-1, and decreased adiponectin, suggesting that impaired glucose metabolism promotes low-grade inflammation (62). Some studies have demonstrated that activation of chronic inflammation and oxidative stress can increase the risk of ASCVD. For example, a 12-year follow-up study of 270,000 women (average age 63) found that the high inflammation group (elevated CRP and fibrinogen) had a 1.5-fold increased risk of cardiovascular events (63). Therefore, we explored the mediation effect of inflammation and oxidative stress on the association of TyG-ABSI with ASCVD. Our results showed that inflammation-related indices (CRP, SIRI, NLR, and MLR) and oxidative stress-related indicators (GGT and UA) had a significant medication effect, indicating the significance of anti-inflammation and anti-oxidative stress on lowering the prevalence of ASCVD. We emphasize that due to the cross-sectional design, this does not confirm a causal mediation pathway. In addition, reverse causation, where prevalent ASCVD elevates both TyG-ABSI and inflammatory markers, remains a plausible alternative explanation for these observed associations. In the near future, more prospective cohort studies should be performed to vadilate thses association.

Currently, nutrition status for patients with ASCVD is significant in reducing the health impact of ASCVD (64). Intake of high levels of monounsaturated fatty acids, such as ω-3, polyphenols such as resveratrol, flavonoids, carotenoids, phytosterols, and soluble and insoluble fibers, could lower the ASCVD risk (65, 66). Dietary patterns represent the overall dietary structure rather than focusing on individual nutrients. Some studies have demonstrated that dietary pattern adjustment may be a more effective way to reduce the ASCVD events compared to only focusing on adjusting the intake level of individual nutrients. In a study of Pilla et al. (67), 102 patients with hypertension and type 2 diabetes were randomly assigned to receive either a modified DASH diet (DASH4D) + low sodium (1,500 mg/day) or a Western diet + high sodium (3,700 mg/day), and thctive way to reduce the risk of ASCVD compared to only focusing on adjusting the ineir results showed that DASH4D + low sodium significantly reduced systolic blood pressure (−4.6 mmHg) and diastolic blood pressure (−2.3 mmHg), superior to the Western diet group. Additionally, A study of 1,034 Swedish adults found that sticking to the Mediterranean diet between the ages of 40 and 60 can lower carotid plaque (OR = 0.72) and carotid intima-media thickness (CIMT) at age 60 (68). Therefore, in this study, we assessed the interaction effect of four dietary patterns (aMed, DASH, DII, and HEI-2020) on the association of TyG-ABSI with ASCVD. Our results revealed that participants with high intake levels of aMed, DASH, and HEI-2020 had lower ASCVD prevalence compared to those with low intake levels of aMed, DASH, and HEI-2020, indicating aMed, DASH, and HEI-2020 had a significant interaction effect.

Specifically, the interaction effect of the Mediterranean and DASH diets may be partly attributed to their potent anti-inflammatory effects. These diets are abundant in foods like olive oil (rich in monounsaturated fats, notably oleic acid), fatty fish (source of long-chain omega-3 fatty acids EPA and DHA), nuts, and leafy green vegetables (69). These components are known to downregulate the production of pro-inflammatory cytokines (e.g., TNF-α, IL-6) and inhibit the NF-κB signaling pathway, a master regulator of inflammation (70, 71). This systemic anti-inflammatory state is crucial for protecting the vascular endothelium and decreasing the risk of ASCVD events. In parallel, the HEI-2020, which emphasizes the intake of fruits, vegetables, and whole grains, confers a high load of dietary antioxidants, such as vitamins C and E, carotenoids, and flavonoids (72). These compounds counteract oxidative stress by neutralizing reactive oxygen species (ROS) and enhancing endogenous antioxidant defenses. Oxidative stress is a key driver of endothelial dysfunction (73); thus, the antioxidant capacity of a high-quality diet, as captured by HEI-2020, plays a critical role in mitigating the metabolic disturbances that contribute to high ASCVD events (73). While the DII provides a direct aggregate measure of dietary inflammation, the aMed, DASH, and HEI-2020 represent dietary frameworks that naturally incorporate these anti-inflammatory and antioxidant principles.

Our study has several strengths. First, this study reveals that the combined effect of TyG and ABSI is better than the individual effects of TyG and ABSI on ASCVD. Second, this study shows the moderate diagnostic efficacies of TyG-ABSI for ASCVD compared to TyG-related markers (TyG, TyG-BMI, TyG-WC, TyG-MHtR). Second, various potential confounding factors, such as basic population characteristics and some clinical indicators, are adjusted to reduce the effect of these covariates on this relationship. Finally, this study presents that inflammation and oxidative stress have remarkable mediation effects on the association between TyG-ABSI and ASCVD.

In addition, there are some limitations to our study. First, as a cross-sectional study, this prevents any conclusion regarding causality or temporal direction. Specifically, the mediation analysis assumes a directional pathway from exposure to outcome through the mediator, but our data cannot verify this sequence. Our analysis identified an association between TyG-ABSI and ASCVD and further revealed that inflammation and oxidative stress appear to be linked to both TyG-ABSI and ASCVD in a manner consistent with a mediating role. However, the cross-sectional nature of this study is a critical limitation that precludes causal inference. Therefore, these analyses should be viewed strictly as exploratory and hypothesis-generating. In addition, we cannot determine the direction of the relationship since it is equally plausible that subclinical or established ASCVD leads to metabolic dysfunction and elevated inflammatory markers (reverse causation). Second, in our retrospective cross-sectional study, some potential confounding factors, such as unmeasured genetic predispositions (e.g., polygenic risk scores for ASCVD), were not adjusted, which may introduce bias in the observed associations. Third, dietary intake in our study was self-reported and may be subject to potential recall bias, and causal inference regarding the direction of the relationships between diet, TyG-ABSI, and ASCVD was prevented by this cross-sectional design. Fourth, we do not further explore the potential biological mechanisms of the modification effect of dietary patterns on the association. Finally, the study participants were recruited from a single center in China, which may limit the generalizability of our findings to other populations with different genetic backgrounds, dietary habits, cultural contexts, and healthcare practices, which may result in these findings being limited in their generalization. The associations we observed between TyG-ABSI and ASCVD warrant validation in more diverse and multi-ethnic cohorts to confirm their universal applicability. Therefore, in the future, well-designed prospective multi-ethnic longitudinal cohort studies will be prioritized to establish causal relationships and elucidate the underlying biological mechanisms.

## 5 Conclusion

Our cross-sectional study reveals a significant positive dose-response linear relationship between TyG-ABSI and ASCVD. Additionally, compared to TyG, ABSI, and TyG-BMI, TyG-ABSI demonstrates better diagnostic capacity for ASCVD diagnosis. In addition, the CRP, SIRI, NLR, MLR, GGT, and UA significantly mediated the association between TyG-ABSI and ASCVD. However, these inflammation and oxidative indicators should be viewed strictly as exploratory and hypothesis-generating for a mediating role, due to our cross-sectional design. Dietary patterns, such as aMed, HEI-2020, DASH, may have modifying effects on the association. In summary, this study elucidates the positive association of the combined effect of TyG and ABSI with ASCVD, advancing the importance of anti-inflammation and anti-oxidative stress in lowering the events of cardiovascular disease.

## Data Availability

The original contributions presented in the study are included in the article/Supplementary material, further inquiries can be directed to the corresponding author.

## References

[1] Wargny M Goronflot T Piriou PG Pouriel M Bastien A Prax J . Persistent gaps in the implementation of lipid-lowering therapy in patients with established atherosclerotic cardiovascular disease: a French nationwide study. Diabetes Metab. (2025) 51:101638. doi: 10.1016/j.diabet.2025.101638 40101894

[2] Kong X Cai Y Li Y Wang P. Causal relationship between apolipoprotein B and risk of atherosclerotic cardiovascular disease: a mendelian randomization analysis. Health Inf Sci Syst. (2025) 13:13. doi: 10.1007/s13755-024-00323-5 39758974 PMC11698695

[3] Alkandari H Jayyousi A Shalaby A Alromaihi D Subbarao G ElMohamedy H . Prevalence of atherosclerotic cardiovascular disease in people with type 2 diabetes in the Gulf Region: results from the PACT-MEA study. Public Health. (2025) 242:21–7. doi: 10.1016/j.puhe.2025.02.026 40020490

[4] Moorthy S Chen Z Zhang T Ponnana SR Sirasapalli SK Shivanantham K . The built environment and adverse cardiovascular events in US veterans with cardiovascular disease. Sci Total Environ. (2025) 980:179596. doi: 10.1016/j.scitotenv.2025.179596 40319806

[5] Gui Z Chen X Wang D Chen Z Liu S Yu G . Inflammatory and metabolic markers mediate the association of hepatic steatosis and fibrosis with 10-year ASCVD risk. Ann Med. (2025) 57:2486594. doi: 10.1080/07853890.2025.2486594 40189927 PMC11980196

[6] Yan L Zhou Z Wu X Qiu Y Liu Z Luo L . Association between the changes in the estimated glucose disposal rate and new-onset cardiovascular disease in middle-aged and elderly individuals: a nationwide prospective cohort study in China. Diabetes Obes Metab. (2025) 27:1859–67. doi: 10.1111/dom.16179 39762991 PMC11885094

[7] Wu L Bao X Xu J Ma L Kang L Zhang R. The triglyceride-glucose index positively associates with the prevalence and severity of coronary heart disease in patients among hypertension. Sci Rep. (2025) 15:19571. doi: 10.1038/s41598-025-03948-y 40467641 PMC12137601

[8] Rayner JJ Abdesselam I Pan J Lewis AJM Rider OJ. Obesity and heart failure: exploring the cardiometabolic axis. Cardiovasc Res. (2025) 121:1173–86. doi: 10.1093/cvr/cvaf090 40458047 PMC12310286

[9] Teixeira PP Xu YY Aravkin A Zheng P Force LM Kocarnik J . A burden of proof study of the effects of exposure to high fasting plasma glucose on the risk of seven types of cancer. Sci Rep. (2025) 15:28859. doi: 10.1038/s41598-025-13045-9 40775253 PMC12331999

[10] Zhu J Duan Y Li Y Zhou Y Lu Z Chen N . Insulin resistance as a mediator of the association between obesity, high-intensity binge drinking, and liver enzyme abnormalities in young and middle-aged adults: a cross-sectional study. Front Nutr. (2025) 12:1554392. doi: 10.3389/fnut.2025.1554392 40777174 PMC12328162

[11] Hsu KM Hsieh YP Chang YJ Tsai SM Chiu PF. Triglyceride-glucose index predicts the mortality risk among incident peritoneal dialysis patients in a cohort study. Sci Rep. (2025) 15:35283. doi: 10.1038/s41598-025-19171-8 41068227 PMC12511594

[12] Hu S Yan H Sun Y Wang D Zeng H Cui G. Triglyceride glucose index in patients with acute coronary syndrome undergoing percutaneous coronary intervention predicts cardiovascular events: a cohort study. Cardiovasc Diabetol. (2025) 24:380. doi: 10.1186/s12933-025-02937-9 41034995 PMC12487205

[13] Zhu F Gan W Mao H Nie S Zeng X Chen W. Association between the triglyceride-glucose index and major adverse cardiovascular events in patients with chronic kidney disease stages 3–4. Sci Rep. (2025) 15:28538. doi: 10.1038/s41598-025-14057-1 40764800 PMC12325913

[14] Feng B Zhao Y Xu W Meng X Li Y Xia C . Triglyceride-glucose index as a novel prognostic biomarker for coronary artery disease: evidence from a large-scale prospective cohort study. Front Endocrinol. (2025) 16:1653948. doi: 10.3389/fendo.2025.1653948 41019316 PMC12460115

[15] Suaárez-González KD Solis-Manzano AM Padilla-Samaniego MV Sandoval-Tamayo VP Morales-Caluña ER. Relationship between diet, sociodemographic factors, and body composition in students from UNEMI and ESPOCH. Front Public Health. (2025) 13:1621661. doi: 10.3389/fpubh.2025.1621661 40777649 PMC12330285

[16] Lee TL Lin FJ Yeh CF Hsiao YC Yang KC Hsuan CF . Evaluating the potential of waist-to-BMI ratio, a body shape index, and other anthropometric parameters in predicting cardiovascular disease mortality: evidence from NHANES III. BMC Public Health. (2025) 25:1828. doi: 10.1186/s12889-025-22944-5 40382539 PMC12085018

[17] Zheng X Zhang W Yang F Wang L Yu B Liang B. Evaluative performance of TyG-ABSI versus traditional indices in relation to cardiovascular disease and mortality: evidence from the U.S. NHANES. Cardiovasc Diabetol. (2025) 24:344. doi: 10.1186/s12933-025-02902-6 40841630 PMC12372269

[18] Yue Y Li P Sun Z Murayama R Li Z Hashimoto K . Association of novel triglyceride-glucose-related indices with incident stroke in early-stage cardiovascular-kidney-metabolic syndrome. Cardiovasc Diabetol. (2025) 24:301. doi: 10.1186/s12933-025-02854-x 40707904 PMC12291248

[19] Chen M Gao Q Zhu X Zhang J Xia R Zhang Q. Dietary inflammatory index and depressive symptoms as mediators between social disadvantage and accelerated phenotypic aging. J Affect Disord. (2025) 391:119995. doi: 10.1016/j.jad.2025.119995 40749804

[20] Alomari WD Almoraie NM. Ultra-processed food intake and its association with obesity risk factors, mediterranean diet, and nutrient intake of adults. Front Nutr. (2025) 12:1577431. doi: 10.3389/fnut.2025.1577431 40777178 PMC12328165

[21] Puértolas-Balint F Prasoodanan PKV Holmberg SM Schröder BO. Disentangling the impact of obesity, diet, host factors, and microbiota on small intestinal antimicrobial peptide expression. Gut Microbes. (2025) 17:2536095. doi: 10.1080/19490976.2025.2536095 40760765 PMC12326570

[22] Cai Y Yang M Ma S Zhang J Huang B Yu B . A meta-analysis of the prognostic value of the TyG index in heart failure. Front Endocrinol. (2025) 16:1463647. doi: 10.3389/fendo.2025.1463647 40778275 PMC12328152

[23] Papagiannopoulos CK Markozannes G Chalitsios CV Christakoudi S Gunter MJ Dossus L . Sex-stratified metabolic signatures of adiposity indices and their associations with clinical biomarkers in the UK Biobank. EBioMedicine. (2025) 119:105868. doi: 10.1016/j.ebiom.2025.105868 40768833 PMC12789707

[24] Li J Chen XL Ou-Yang XL Zhang XJ Li Y Sun SN . Association of tea consumption with all-cause/cardiovascular disease mortality in the chronic kidney disease population: an assessment of participation in the national cohort. Ren Fail. (2025) 47:2449578. doi: 10.1080/0886022X.2025.2449578 39806767 PMC11734394

[25] Hao W Shan YF Kimura T Ukawa S Ohira H Okabayashi S . Effect modification by dietary patterns in the relationship between slow gait and incident depressive symptoms: a 6-year cohort study of older Japanese adults (NISSIN Project). Front Nutr. (2025) 12:1698581. doi: 10.3389/fnut.2025.1698581 41450553 PMC12728573

[26] Li Y Gu W Hao W Xu Y Li K Zhao Y . Associations of four important dietary pattern scores, micronutrients with sarcopenia and osteopenia in adults: results from the National Health and Nutrition Examination Survey. Front Nutr. (2025) 12:1583795. doi: 10.3389/fnut.2025.1583795 40771224 PMC12325016

[27] Al Shamsi HSS Gardener SL Rainey-Smith SR Sohrabi HR Taddei K Masters CL . The role of diet in moderating the relationship between symptoms of depression and brain amyloid load. Alzheimers Dement. (2025) 21:e70560. doi: 10.1002/alz.70560 40775676 PMC12331443

[28] Meng X Chen X Zhang B Wang J. Comparative analysis of dietary pattern indices and their associations with chronic kidney disease: a comprehensive analysis of NHANES data (2000–2020). Ren Fail. (2025) 47:2540565. doi: 10.1080/0886022X.2025.2540565 40765017 PMC12329849

[29] Greaney C McCarthy E O'Brien L Tecklenborg S Howlett C Cronin K . Interrelationships between diet quality and health-related quality of life in Irish adults living with cystic fibrosis. Eur J Nutr. (2025) 64:248. doi: 10.1007/s00394-025-03766-y 40705145 PMC12289792

[30] Zhuge Z Zheng K Ji X Wang X Zhang X. Association between the oxidative balance score and testosterone deficiency in males: a cross-sectional study. Front Nutr. (2025) 12:1577823. doi: 10.3389/fnut.2025.1577823 40771217 PMC12325012

[31] Hong T Lian Z Zhang C Zhang W Ye Z. Hypertension modifies the association between serum Klotho and chronic kidney disease in US adults with diabetes: a cross-sectional study of the NHANES 2007–2016. Ren Fail. (2025) 47:2498089. doi: 10.1080/0886022X.2025.2498089 40324899 PMC12054556

[32] Liu C Yang J Li H Deng Y Dong S He P . Association between life's essential 8 and diabetic kidney disease: a population-based study. Ren Fail. (2025) 47:2454286. doi: 10.1080/0886022X.2025.2454286 40064556 PMC11894740

[33] Li S Liu HH Zhang Y Zhang M Zhang HW Zhu CG . Association of triglyceride glucose-derived indices with recurrent events following atherosclerotic cardiovascular disease. J Obes Metab Syndr. (2024) 33:133–42. doi: 10.7570/jomes23055 38714326 PMC11224920

[34] Xia X Chen S Tian X Xu Q Zhang Y Zhang X . Association of triglyceride-glucose index and its related parameters with atherosclerotic cardiovascular disease: evidence from a 15-year follow-up of Kailuan cohort. Cardiovasc Diabetol. (2024) 23:208. doi: 10.1186/s12933-024-02290-3 38898520 PMC11188278

[35] Hou Q Qi Q Han Q Yu J Wu J Yang H . Association of the triglyceride-glucose index with early-onset atherosclerotic cardiovascular disease events and all-cause mortality: a prospective cohort study. Cardiovasc Diabetol. (2024) 23:149. doi: 10.1186/s12933-024-02249-4 38685099 PMC11059708

[36] Elsabaawy M Naguib M Abuamer A Shaban A. Comparative application of MAFLD and MASLD diagnostic criteria on NAFLD patients: insights from a single-center cohort. Clin Exp Med. (2025) 25:36. doi: 10.1007/s10238-024-01553-3 39808219 PMC11732950

[37] Antonio-Villa NE Juárez-Rojas JG Posadas-Sánchez R Reyes-Barrera J Medina-Urrutia A. Visceral adipose tissue is an independent predictor and mediator of the progression of coronary calcification: a prospective sub-analysis of the GEA study. Cardiovasc Diabetol. (2023) 22:81. doi: 10.1186/s12933-023-01807-6 37013573 PMC10071707

[38] Wu Z Lan Y Wu D Chen S Jiao R Wu S. Arterial stiffness mediates insulin resistance-related risk of atherosclerotic cardiovascular disease: a real-life, prospective cohort study. Eur J Prev Cardiol. (2025) zwaf030. doi: 10.1093/eurjpc/zwaf030. [Epub ahead of print]. 39847612

[39] Rudolfsen JH Vukmirica J Johansen P Røder KL Mortensen MB. Impact of systemic inflammation on healthcare resource utilisation and cost in patients with atherosclerotic cardiovascular disease and chronic kidney disease. J Med Econ. (2025) 28:1298–306. doi: 10.1080/13696998.2025.2542024 40758484

[40] Wu X Zhang H Liu H. Systemic immune-inflammation index and systemic inflammation response index levels are associated with coronary heart disease prevalence in the asthmatic population: a cross-sectional analysis of the NHANES 2011–2018. Front Public Health. (2025) 13:1514016. doi: 10.3389/fpubh.2025.1514016 40697833 PMC12279799

[41] Zhang A Liu Y Mei L Zhang Y Pan Y Zhu B. Association between serum gamma-glutamyl transferase levels and polyvascular atherosclerotic plaques and stenosis: a cross-sectional study. Cerebrovasc Dis. (2025) 1−23. doi: 10.1159/000547701. [Epub ahead of print]. 40759090

[42] Stephenson SS Kravchenko G Gawron-Skarbek A Kostka T Sołtysik BK. Association between immuno-nutritional biomarkers and mortality in hospitalized geriatric population. Front Immunol. (2025) 16:1692551. doi: 10.3389/fimmu.2025.1692551 41357173 PMC12676944

[43] Li Y Yan X Xu Y Pope R Spector TD Falchi M . Higher adherence to (poly)phenol-rich diet is associated with lower CVD risk in the TwinsUK cohort. BMC Med. (2025) 23:645. doi: 10.1186/s12916-025-04481-5 41299455 PMC12659045

[44] Tang Q Wang Y Luo Y. An interpretable machine learning model with demographic variables and dietary patterns for ASCVD identification: from U.S. NHANES 1999–2018. BMC Med Inform Decis Mak. (2025) 25:105. doi: 10.1186/s12911-025-02937-5 40033349 PMC11874124

[45] Peng W Li Z Fu N. Association between eGDR and MASLD and liver fibrosis: a cross-sectional study based on NHANES 2017–2023. Front Med. (2025) 12:1579879. doi: 10.3389/fmed.2025.1579879 40520791 PMC12162473

[46] Russo S Kwiatkowski M Govorukhina N Bischoff R Melgert BN. Meta-inflammation and metabolic reprogramming of macrophages in diabetes and obesity: the importance of metabolites. Front Immunol. (2021) 12:746151. doi: 10.3389/fimmu.2021.746151 34804028 PMC8602812

[47] Alhowail A Aldawsari MF Aldubayan M. Comparative analysis of pioglitazone and tirzepatide on body weight, glucose levels, neuroinflammation, and oxidative stress in diabetic rats. Drug Des Devel Ther. (2025) 19:6605–18. doi: 10.2147/DDDT.S525690 40771859 PMC12325112

[48] Alsaedi AQ Nader MA El-Kashef DH Abdelmageed ME. Mangiferin mitigates dexamethasone-induced insulin resistance in rats: insight into vascular dysfunction and hepatic steatosis. Front Pharmacol. (2025) 16:1572758. doi: 10.3389/fphar.2025.1572758 40406487 PMC12095298

[49] Pavanello C Ruscica M Castiglione S Mombelli GG Alberti A Calabresi L . Triglyceride-glucose index: carotid intima-media thickness and cardiovascular risk in a European population. Cardiovasc Diabetol. (2025) 24:17. doi: 10.1186/s12933-025-02574-2 39806381 PMC11731386

[50] Gu M Zhang D Wu Y Li X Liang J Su Y . Association between brachial-ankle pulse wave velocity, obesity-related indices, and the 10-year incident risk score of atherosclerotic cardiovascular disease: the rural Chinese cohort study. Nutr Metab Cardiovasc Dis. (2025) 35:103791. doi: 10.1016/j.numecd.2024.103791 39672744

[51] Cho YR Ann SH Won KB Park GM Kim YG Yang DH . Association between insulin resistance, hyperglycemia, and coronary artery disease according to the presence of diabetes. Sci Rep. (2019) 9:6129. doi: 10.1038/s41598-019-42700-1 31477741 PMC6718672

[52] Ladeira LLC Nascimento GG Leite FRM Alves-Costa S Barbosa JMA Alves CMC . Obesity, insulin resistance, caries, and periodontitis: syndemic framework. Nutrients. (2023) 15:3512. doi: 10.3390/nu15163512 37630703 PMC10458482

[53] Abdallah H Khalil M Awada E Lanza E Di Ciaula A Portincasa P. Metabolic dysfunction-associated steatotic liver disease (MASLD). Assessing metabolic dysfunction, cardiovascular risk factors, and lifestyle habits. Eur J Intern Med. (2025) 138:101–11. doi: 10.1016/j.ejim.2025.05.018 40436716

[54] Makhmudova U Wild B Williamson A Steinhagen-Thiessen E Langenberg C Eils R . Visceral adipose tissue, aortic distensibility and atherosclerotic cardiovascular risk across body mass index categories. Eur J Prev Cardiol. (2025) zwaf447. doi: 10.1093/eurjpc/zwaf447. [Epub ahead of print]. 40680099

[55] Zhao YC Wu SQ Li JK Sun ZH Zhang BK Fu R . Predictive value of the combined triglyceride-glucose and frailty index for cardiovascular disease and stroke in two prospective cohorts. Cardiovasc Diabetol. (2025) 24:318. doi: 10.1186/s12933-025-02880-9 40759963 PMC12323107

[56] Pithová P Cichrová M Kvapil M Hubáček JA Dlouhá D Pitha J. Determinants of vascular impairment in type 1 diabetes-impact of sex and connexin 37 gene polymorphism: a cross-sectional study. Cardiovasc Diabetol. (2024) 23:309. doi: 10.1186/s12933-024-02401-0 39175027 PMC11342627

[57] Zhou S Guo R. Understanding triglyceride glucose-body mass index: implications for diabetes and cardiovascular disease management. Front Endocrinol. (2025) 16:1675270. doi: 10.3389/fendo.2025.1675270 41089283 PMC12515649

[58] Neumann K Mawandji NBS Schereider IRG de Oliveira EC Vieira JM Bolsoni-Lopes A . A refined carbohydrate-rich diet reduces vascular reactivity through endothelial oxidative stress and increased nitric oxide: the involvement of inducible nitric oxide synthase. Nutrients. (2025) 17:2395. doi: 10.3390/nu17152395 40805981 PMC12348616

[59] Biolo G Di Girolamo FG Breglia A Chiuc M Baglio V Vinci P . Inverse relationship between “a body shape index” (ABSI) and fat-free mass in women and men: insights into mechanisms of sarcopenic obesity. Clin Nutr. (2015) 34:323–7. doi: 10.1016/j.clnu.2014.03.015 24814384

[60] Liu X Ma Q Feng Y Wang F Wang W Wang J . Potato resistant starch improves type 2 diabetes by regulating inflammation, glucose and lipid metabolism and intestinal microbial environment. Int J Biol Macromol. (2024) 281:136389. doi: 10.1016/j.ijbiomac.2024.136389 39389507

[61] Carmona-Maurici J Ricart-Jané D Viñas A López-Tejero MD Eskubi-Turró I Miñarro A . Circulating miRNAs as biomarkers of subclinical atherosclerosis associated with severe obesity before and after bariatric surgery. Obes Facts. (2024) 17:602–12. doi: 10.1159/000541175 39236703 PMC11661843

[62] Manga J Odell N Khambule L Harishun S Mohamed F. Hyperglycemia in pregnancy: outcomes and diagnostic accuracy of combined modalities. Clin Med. (2025) 25:100495. doi: 10.1016/j.clinme.2025.100495 PMC12395516 40774546

[63] Lin Y Louca P Bowyer RCE Kouraki A Rossi N Ni Lochlainn M . Common inflammatory proteins linking frailty and area-level deprivation as key drivers of cardiovascular risk in women. Commun Med. (2025) 5:301. doi: 10.1038/s43856-025-01012-4 40684041 PMC12276345

[64] Berisha H Hattab R Comi L Giglione C Migliaccio S Magni P. Nutrition and lifestyle interventions in managing dyslipidemia and cardiometabolic risk. Nutrients. (2025) 17:776. doi: 10.3390/nu17050776 40077646 PMC11902110

[65] Riley TM Sapp PA Kris-Etherton PM Petersen KS. Effects of saturated fatty acid consumption on lipoprotein (a): a systematic review and meta-analysis of randomized controlled trials. Am J Clin Nutr. (2024) 120:619–29. doi: 10.1016/j.ajcnut.2024.06.019 38964657

[66] Yu W Zhao Y Ilyas I Wang L Little PJ Xu S. The natural polyphenol fisetin in atherosclerosis prevention: a mechanistic review. J Pharm Pharmacol. (2025) 77:206–21. doi: 10.1093/jpp/rgae053 38733634

[67] Pilla SJ Yeh HC Mitchell CM Miller ER 3rd Oh S White K . Dietary patterns, sodium reduction, and blood pressure in type 2 diabetes: the DASH4D randomized clinical trial. JAMA Intern Med. (2025) 185:937–46. doi: 10.1161/cir.151.suppl_1.022 40489102 PMC12150227

[68] Almevall AD Wennberg P Liv P Nyman E Lindvall K Norberg M . Midlife mediterranean diet is associated with subclinical carotid atherosclerosis in late midlife. Eur J Prev Cardiol. (2025) 32:1614–28. doi: 10.1093/eurjpc/zwaf155 40100758

[69] Cherian L Agarwal P Agrawal S James BD Yang D Wagner M . Dietary patterns associated with risk of intracranial atherosclerosis in older adults with hypertension or myocardial infarction. Neurology. (2025) 105:e214147. doi: 10.1212/WNL.0000000000214147 41066721 PMC12509707

[70] Smidowicz A Regula J. Effect of nutritional status and dietary patterns on human serum C-reactive protein and interleukin-6 concentrations. Adv Nutr. (2015) 6:738–47. doi: 10.3945/an.115.009415 26567198 PMC4642421

[71] González-Rodríguez M Ait Edjoudi D Cordero-Barreal A Farrag M Varela-García M Torrijos-Pulpón C . Oleocanthal, an antioxidant phenolic compound in extra virgin olive oil (EVOO): a comprehensive systematic review of its potential in inflammation and cancer. Antioxidants. (2023) 12:2112. doi: 10.3390/antiox12122112 38136231 PMC10741130

[72] Sualeheen A Tan SY Daly RM Georgousopoulou E Roberts SK George ES. Higher diet quality is associated with a lower prevalence of MASLD and adverse health outcomes: insights from NHANES 2005 to 2020. Eur J Nutr. (2025) 64:289. doi: 10.1007/s00394-025-03809-4 41051618 PMC12500818

[73] D'Imperio S Monasky MM Micaglio E Negro G Pappone C. Impact of dietary factors on Brugada syndrome and long QT syndrome. Nutrients. (2021) 13:2482. doi: 10.3390/nu13082482 34444641 PMC8401538

