# Supplementary material for: Association between triglyceride-glucose index-a body shape index and atherosclerotic cardiovascular disease, and the modification effect of dietary patterns
Source: Front Nutr. 2026 Mar 10;13:1682636. doi: 10.3389/fnut.2026.1682636 (PMC13008743; doi:10.3389/fnut.2026.1682636)
Supplement: Supplementary file 1 [file Table_1.docx]

**Table S1.** Some TyG-related indicators for diagnosing the ASCVD

|  | AUC (95%CI) | Cutoff value | Sensitivity | Specificity | NPV | PPV |
| --- | --- | --- | --- | --- | --- | --- |
| TyG | 0.649  (0.630, 0.668) | 4.512 | 0.668 | 0.512 | 0.576 | 0.636 |
| ABSI | 0.629  (0.611, 0.646) | 0.845 | 0.653 | 0.536 | 0.552 | 0.645 |
| TyG-BMI | 0.651  (0.634, 0.668) | 96.673 | 0.674 | 0.541 | 0.589 | 0.664 |
| TyG-WC | 0.680  (0.664, 0.696) | 330.35 | 0.653 | 0.557 | 0.580 | 0.678 |
| TyG-MHtR | 0.664  (0.648, 0.681) | 20.563 | 0.675 | 0.548 | 0.563 | 0.637 |
| TyG-ABSI | 0.718  (0.702, 0.733) | 3.245 | 0.726 | 0.618 | 0.601 | 0.653 |

**Abbreviations:** AUC: area under the curve, NPV: negative predictive value, PPV: positive predictive value. **Notes:** All covariates were adjusted in the model, except for CRP, GGT, UA, SIRI, NLR, and MLR. *P* < 0.05 was regarded as statistically significant.

**TableS2.** Association between inflammation and oxidative stress, and TyG-ABSI

|  | β (95%CI) | *P*-value |
| --- | --- | --- |
| CRP |  |  |
| Continuous | 1.162 (0.786, 1.488) | < 0.001 |
| Q1 | Reference |  |
| Q2 | 0.578 (0.362, 0.804) | < 0.001 |
| Q3 | 0.894 (0.653, 1.248) | < 0.001 |
| Q4 | 1.375 (0.875, 1.879) | < 0.001 |
| *P* for trend | < 0.001 |  |
| SIRI |  |  |
| Continuous | 0.892 (0.567, 1.341) | < 0.001 |
| Q1 | Reference |  |
| Q2 | 0.432 (0.214, 0.748) | < 0.001 |
| Q3 | 0.698 (0.412, 0.987) | < 0.001 |
| Q4 | 1.120 (0.812, 1.569) | < 0.001 |
| *P* for trend | < 0.001 |  |
| NLR |  |  |
| Continuous | 0.571 (0.210, 0.897) | < 0.001 |
| Q1 | Reference |  |
| Q2 | 0.235 (-0.032, 0.614) | 0.061 |
| Q3 | 0.489 (0.231, 0.785) | < 0.001 |
| Q4 | 0.712 (0.413, 1.087) | < 0.001 |
| *P* for trend | < 0.001 |  |
| MLR |  |  |
| Continuous | 0.701 (0.336, 1.102) | < 0.001 |
| Q1 | Reference |  |
| Q2 | 0.389 (0.218, 0.628) | < 0.001 |
| Q3 | 0.658 (0.410, 0.914) | < 0.001 |
| Q4 | 0.928 (0.601, 1.319) | < 0.001 |
| *P* for trend | < 0.001 |  |
| GCT |  |  |
| Continuous | 0.987 (0.676, 1.338) | < 0.001 |
| Q1 | Reference |  |
| Q2 | 0.562 (0.356, 0.801) | < 0.001 |
| Q3 | 0.762 (0.512, 1.101) | < 0.001 |
| Q4 | 1.256 (1.012, 1.765) | < 0.001 |
| *P* for trend | < 0.001 |  |
| UA |  |  |
| Continuous | 0.745 (0.486, 1.041) | < 0.001 |
| Q1 | Reference |  |
| Q2 | 0.356 (0.189, 0.612) | < 0.001 |
| Q3 | 0.654 (0.312, 0.892) | < 0.001 |
| Q4 | 1.181 (0.683, 1.901) | < 0.001 |
| *P* for trend | < 0.001 |  |

**Abbreviation:** OR: odds ratio. **Notes:** Q1 was regarded as the reference group. All covariates were adjusted in the model, except for CRP, GGT, UA, SIRI, NLR, and MLR. *P* < 0.05 was regarded as statistically significant.

**TableS3.** Association between inflammation and oxidative stress, and ASCVD risk

|  | OR (95%CI) | *P*-value |
| --- | --- | --- |
| CRP |  |  |
| Continuous | 2.362 (1.386, 4.134) | < 0.001 |
| Q1 | Reference |  |
| Q2 | 1.212 (0.672, 1.804) | 0.235 |
| Q3 | 1.764 (1.105, 2.769) | 0.003 |
| Q4 | 3.075 (1.816, 5.218) | < 0.001 |
| *P* for trend | < 0.001 |  |
| SIRI |  |  |
| Continuous | 1.729 (1.167, 3.421) | 0.001 |
| Q1 | Reference |  |
| Q2 | 1.183 (0.713, 1.762) | 0.316 |
| Q3 | 1.527 (1.192, 2.002) | 0.001 |
| Q4 | 2.421 (1.601, 3.487) | < 0.001 |
| *P* for trend | < 0.001 |  |
| NLR |  |  |
| Continuous | 1.332 (1.098, 1.863) | 0.005 |
| Q1 | Reference |  |
| Q2 | 0.984 (0.861, 1.301) | 0.387 |
| Q3 | 1.374 (1.103, 1.765) | 0.002 |
| Q4 | 2.021 (1.342, 2.874) | < 0.001 |
| *P* for trend | < 0.001 |  |
| MLR |  |  |
| Continuous | 1.612 (1.145, 2.692) | 0.002 |
| Q1 | Reference |  |
| Q2 | 1.189 (1.054, 1.682) | 0.032 |
| Q3 | 1.511 (1.219, 2.112) | 0.001 |
| Q4 | 2.328 (1.532, 3.135) | < 0.001 |
| *P* for trend | < 0.001 |  |
| GCT |  |  |
| Continuous | 2.026 (1.312, 4.012) | < 0.001 |
| Q1 | Reference |  |
| Q2 | 1.362 (1.156, 1.886) | 0.001 |
| Q3 | 1.745 (1.321, 2.312) | < 0.001 |
| Q4 | 2.535 (1.763, 4.496) | < 0.001 |
| *P* for trend | < 0.001 |  |
| UA |  |  |
| Continuous | 1.641 (1.186, 2.342) | 0.001 |
| Q1 | Reference |  |
| Q2 | 1.256 (1.069, 1.812) | 0.029 |
| Q3 | 1.589 (1.282, 2.095) | < 0.001 |
| Q4 | 2.287 (1.696, 4.034) | < 0.001 |
| *P* for trend | < 0.001 |  |

**Abbreviation:** OR: odds ratio. **Notes:** Q1 was regarded as the reference group. All covariates were adjusted in the model, except for CRP, GGT, UA, SIRI, NLR, and MLR. *P* < 0.05 was regarded as statistically significant.

Table S4. Association between TyG-ABSI and ASCVD after additionally adjusting for women's menopausal status

|  | OR (95%CI) | *P*-value |
| --- | --- | --- |
| Model Ⅰ |  |  |
| Continuous | 2.936 (2.140, 4.851) | < 0.001 |
| Q1 | Reference |  |
| Q2 | 1.374 (1.191, 1.623) | < 0.001 |
| Q3 | 1.832 (1.342, 2.816) | < 0.001 |
| Q4 | 3.212 (2.365, 4.627) | < 0.001 |
| *P* for trend | < 0.001 |  |
| Model Ⅱ |  |  |
| Continuous | 2.753 (1.812, 4.560) | < 0.001 |
| Q1 | Reference |  |
| Q2 | 1.286 (1.162, 1.429) | < 0.001 |
| Q3 | 1.625 (1.315, 2.352) | < 0.001 |
| Q4 | 2.815 (2.138, 4.317) | < 0.001 |
| *P* for trend | <0.001 |  |
| Model Ⅲ |  |  |
| Continuous | 2.414 (1.627, 4.213) | < 0.001 |
| Q1 | Reference |  |
| Q2 | 1.230 (1.103, 1.398) | < 0.001 |
| Q3 | 1.557 (1.218, 2.182) | < 0.001 |
| Q4 | 2.516 (1.986, 3.987) | < 0.001 |
| *P* for trend | < 0.001 |  |

**Abbreviations:** Q: quartile, OR: odds ratio. **Notes:** Q1 was regarded as the reference group. All covariates were adjusted in the model, except for CRP, GGT, UA, SIRI, NLR, and MLR. *P* < 0.05 was regarded as statistically significant.

Table S5. Association between TyG-ABSI and ASCVD after additionally adjusting for medication use (anti-diabetics, anti-inflammatories, antihypertensives, and statins).

|  | OR (95%CI) | *P*-value |
| --- | --- | --- |
| Model Ⅰ |  |  |
| Continuous | 2.848 (2.213, 4.963) | < 0.001 |
| Q1 | Reference |  |
| Q2 | 1.341 (1.214, 1.597) | < 0.001 |
| Q3 | 1.975 (1.586, 2.887) | < 0.001 |
| Q4 | 3.065 (2.287, 5.165) | < 0.001 |
| *P* for trend | < 0.001 |  |
| Model Ⅱ |  |  |
| Continuous | 2.712 (1.935, 4.715) | < 0.001 |
| Q1 | Reference |  |
| Q2 | 1.262 (1.173, 1.575) | < 0.001 |
| Q3 | 1.813 (1.403, 2.636) | < 0.001 |
| Q4 | 2.846 (2.049, 4.847) | < 0.001 |
| *P* for trend | <0.001 |  |
| Model Ⅲ |  |  |
| Continuous | 2.503 (1.721, 4.326) | < 0.001 |
| Q1 | Reference |  |
| Q2 | 1.221 (1.124, 1.439) | < 0.001 |
| Q3 | 1.625 (1.324, 2.336) | < 0.001 |
| Q4 | 2.637 (1.858, 4.215) | < 0.001 |
| *P* for trend | < 0.001 |  |

**Abbreviations:** Q: quartile, OR: odds ratio. **Notes:** Q1 was regarded as the reference group. All covariates were adjusted in the model, except for CRP, GGT, UA, SIRI, NLR, and MLR. *P* < 0.05 was regarded as statistically significant.

Table S6. Association between TyG-ABSI and ASCVD after standardized TyG and ABSI before TyG-ABSI construction.

|  | OR (95%CI) | *P*-value |
| --- | --- | --- |
| Model Ⅰ |  |  |
| Continuous | 2.521 (2.013, 3.463) | < 0.001 |
| Q1 | Reference |  |
| Q2 | 1.204 (1.104, 1.578) | < 0.001 |
| Q3 | 1.856 (1.325, 2.596) | < 0.001 |
| Q4 | 3.065 (2.287, 4.189) | < 0.001 |
| *P* for trend | < 0.001 |  |
| Model Ⅱ |  |  |
| Continuous | 2.302 (1.416, 3.341) | < 0.001 |
| Q1 | Reference |  |
| Q2 | 1.165 (1.075, 1.424) | < 0.001 |
| Q3 | 1.609 (1.323, 2.408) | < 0.001 |
| Q4 | 2.663 (1.956, 3.543) | < 0.001 |
| *P* for trend | <0.001 |  |
| Model Ⅲ |  |  |
| Continuous | 2.115 (1.314, 3.026) | < 0.001 |
| Q1 | Reference |  |
| Q2 | 1.101 (0.954, 1.313) | < 0.001 |
| Q3 | 1.505 (1.224, 2.036) | < 0.001 |
| Q4 | 2.437 (1.714, 3.219) | < 0.001 |
| *P* for trend | < 0.001 |  |

**Abbreviations:** Q: quartile, OR: odds ratio. **Notes:** Q1 was regarded as the reference group. All covariates were adjusted in the model, except for CRP, GGT, UA, SIRI, NLR, and MLR. *P* < 0.05 was regarded as statistically significant.
